# Programmable RNA Nanostructures Enable Nanopore Detection of Cotranscriptionally Introduced RNA Modifications

**DOI:** 10.1021/acs.nanolett.5c02391

**Published:** 2025-08-04

**Authors:** Iva Mohora, Gerardo Patiño Guillén, Kevin Neis, Julián Valero, Ulrich F. Keyser, Filip Bošković

**Affiliations:** † Cavendish Laboratory, 2152 University of Cambridge, 19 JJ Thomson Avenue, Cambridge CB3 0HE, United Kingdom; ‡ Interdisciplinary Nanoscience Center (iNANO), 1006 Aarhus University, DK-8000 Aarhus, Denmark; § Department of Molecular Biology and Genetics, Aarhus University, DK-8000 Aarhus, Denmark

**Keywords:** solid-state nanopores, nanopore sensing, DNA and RNA nanotechnology, modified nucleotides, RNA labeling

## Abstract

Tracking RNA synthesis and metabolic histories requires cotranscriptional incorporation of modified nucleotides. However, identifying the incorporation of modified nucleotides into nascent RNA remains challenging, particularly for short RNAs. In this work, we developed a method utilizing solid-state nanopores and DNA:RNA nanostructures to detect modified nucleotide incorporation across different RNA length scales, from short to long RNAs transcribed *in vitro*. We identified the incorporation of biotin-modified uridine in short RNAs using a DNA nanostructure coupled with a nanopore readout. As a proof of concept for tracking RNA synthesis, we evaluated the incorporation of azide-modified uridine into long RNAs. To achieve quantitative labeling, we optimized conditions for click chemistry using cyclooctyne-DNA oligonucleotides. Subsequently, we successfully decorated long RNAs with azide-modified uridine and quantified the relative incorporation levels using nanopores. Our study establishes a robust platform for solid-state nanopore characterization of modified nucleotide-containing RNAs, advancing single-molecule analyses of RNA dynamics.

## Graphical abstract

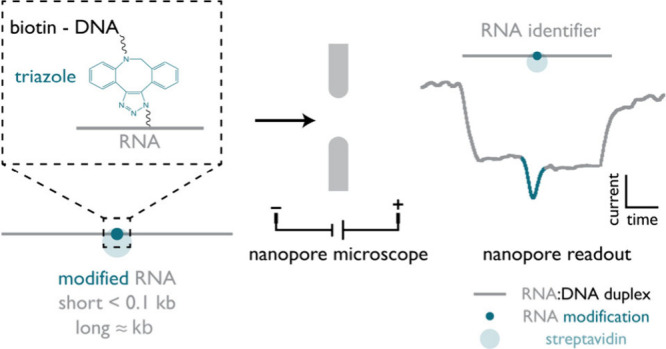

RNA plays a central role in cellular processes, acting as a key intermediary in gene expression and regulation.^1–3^ Its synthesis, turnover, and metabolism are critical for maintaining cellular homeostasis and enabling dynamic responses to environmental cues.^4–6^ In addition to biological RNA metabolism, understanding the stability and metabolism of therapeutic RNAs has become increasingly important.^7,8^

Cotranscriptional labeling involves the incorporation of labeled nucleotide triphosphates directly into RNA during transcription both *in vitro* and *in vivo*.^4,9^ It is a powerful strategy for studying RNA metabolism, allowing temporal tracking of RNA synthesis and turnover. Modified uridine triphosphates (UTPs), particularly 5-position analogues, are widely used in both *in vitro* and *in vivo* RNA synthesis due to their minimal effects on base pairing and negligible disruption of RNA function.^10,11^ This approach enables selective tracking of RNA turnover and transcript-specific changes, providing valuable insights into RNA dynamics. For *in vivo* applications, click group-modified UTPs^9,11,12^ are commonly incorporated during transcription as bioorthogonal handles, while biotin-modified UTPs are preferred for *in vitro* studies.^13^ Azide modifications allow selective enrichment and identification of transcripts via click chemistry,^14^ while biotinylated UTPs facilitate the enrichment of labeled RNAs through biotin–streptavidin pull-down, enabling the distinction of nascent RNAs from preexisting transcripts.^4,13^

Although these chemical handles enable the enrichment of modified RNAs, they require specialized protocols, and thereafter, conventional sequencing methods^15–17^ may not detect or efficiently estimate the level of modifications. While these approaches can reveal transcript age, they often fall short in providing relative quantification of nucleotide modifications.^18^ Enrichment-based methods, such as pull-down assays, are prone to biases arising from variable biotin incorporation, which can affect the strength of transcript-specific interactions and distort transcriptome representation.^4,6^

Single-molecule approaches, particularly solid-state nanopore sensing, offer significant advantages for overcoming these limitations.^16,19,20^ Nanopores facilitate the characterization of native RNA molecules without enzyme-induced biases,^16,21–23^ enabling the detection of a wide range of biomolecules, including DNA, RNA,^21,22,24–26^ and proteins.^27–32^ For short RNA detection, nanopores offer multiplexing capabilities and enhanced specificity, further augmented by DNA:RNA nanotechnology.^33^ Recent advancements have demonstrated the potential of nanopores in identifying native RNA isoforms and diverse RNA species directly from transcriptomes.^16,25,34,35^

Nanopore sensing relies on detecting the physical characteristics of biomolecules as they translocate through a nanopore under an applied electrophoretic force.^36,37^ By binding short RNAs to DNA nanostructures or engineering RNA nanostructures from long RNAs, specific transcripts can be identified based on their distinct nanopore signatures. We explore the detection of incorporation of modified nucleotides in both short and long RNAs utilizing programmable nanostructure-assisted nanopore sensing.

Here, we demonstrate the identification of RNA nanostructures assembled from transcribed short and long RNAs with cotranscriptionally incorporated azide or biotin-modified UTPs, using solid-state nanopore sensing. For azide-UTP-labeled transcripts, we performed a catalyst-free click chemistry reaction with a dibenzocyclooctyne (DBCO)-conjugated oligonucleotide to label the incorporated modifications. Then, we assemble two types of nanostructures depending on RNA length. For short RNA, we use a single-stranded DNA scaffold and anneal a set of short complementary oligonucleotides to form a double-stranded DNA nanostructure with single-stranded overhangs designed to bind the RNA targets. For long RNA (3 kbp), we instead use the RNA itself as the scaffold and hybridize complementary DNA oligonucleotides directly to the transcript. We show that biotin-labeled short RNAs are reliably distinguished from their unmodified counterparts. We transcribe 3 kbp RNA with a 1:10 azide-UTP:UTP ratio, label them with DBCO-C6-DNA, and assemble DNA hybrids to enable semiquantitative nanopore mapping of modification density along full transcripts. Our method provides a complementary approach for the semiquantitative characterization of RNA-containing modified nucleotides, offering new insights into their incorporation and potential applications in tracking RNA metabolic histories.

## Nanopore Detection of Modified RNA across Different Lengths

We analyzed modified RNAs, both short (<100 nucleotides) and long (kilobases) forms with nanopores, by cotranscriptionally introducing modified nucleotides, facilitated by self-assembled nanostructures (Figure 1). We assembled a DNA nanostructure by hybridizing a single-stranded DNA scaffold (2.7 kb, m13mp18 fragment) with short oligonucleotides (≈38 nucleotides) such that it contains two single-stranded DNA overhangs. These overhangs are complementary to unmodified or modified short RNA, and their binding facilitates their downstream detection (Figure 1a, top structure).

**Figure 1:**
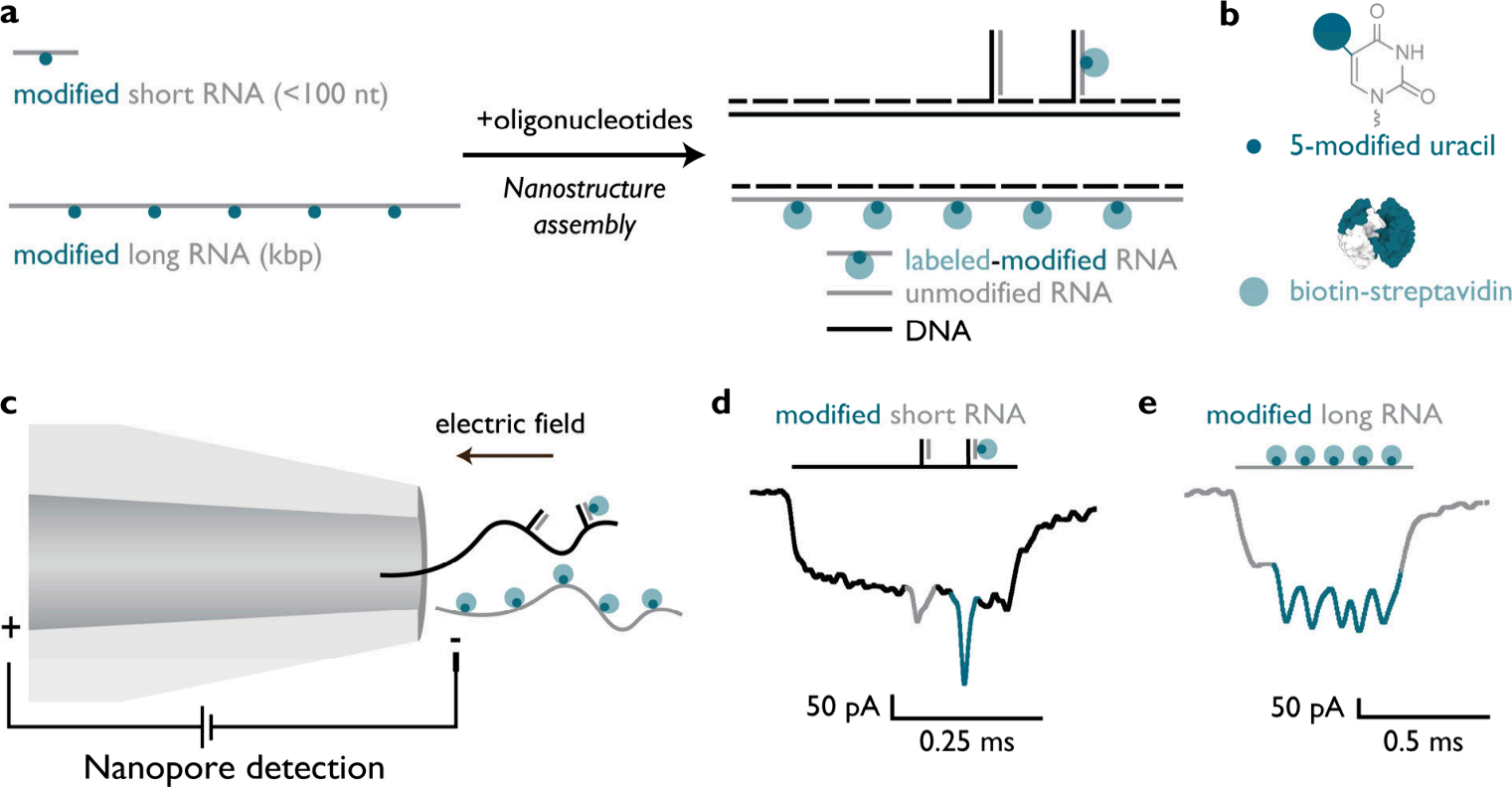
Nanopore detection of short and long modified RNAs. (a) Short, modified RNA (<100 nucleotides) and long, modified RNA (kilobases long) were synthesized by *in vitro* transcription using modified nucleotide triphosphates (RNA in gray and modifications in dark blue). We assembled two nanostructures, the first being the DNA nanostructure containing overhangs to bind short RNAs (unmodified or modified) and the second being the RNA nanostructure assembled with the long, modified RNA serving as a scaffold to which DNA oligonucleotides hybridize (≈38 nucleotides long). Then, the modification signal labeled with biotin was bound to monovalent streptavidin (in light blue). (b) Schematic of the 5-carbon-modified UTPs with biotin or azide used in this study and biotin–streptavidin, with the single biotin binding site colored white. (c) RNA nanostructure consisting of long modified RNA (azide-UTP) or DNA nanostructure carrying short RNAs (biotin-UTP) was electrophoretically translocated through the glass nanopore. Example nanopore events for the DNA nanostructure harboring short, modified RNA or the long, modified RNA nanostructure are shown in panels d and e, respectively.

For long modified RNA, we used the modified RNAs as a scaffold and assembled the nanostructure by hybridizing short oligonucleotides (≈38 nucleotides (Figure 1a, bottom structure)). In order to enable sequential readout of labeled modifications along the long RNA transcript, the molecule is converted from an unpaired single-stranded state into a linear double-stranded duplex. The increased persistence length of the duplex facilitates the linear readout with nanopores.^16,38,39^ For both types of nanostructures, DNA oligonucleotides (≈38 nucleotides) were added in excess to ensure duplex formation. The oligonucleotide length is chosen to ensure the formation of a stable duplex and to limit secondary structure formation. We used the 5-carbon position of uridine in RNA as an example RNA modification site (Figure 1b). Modified UTPs (the detailed structure of modified UTPs is shown in Figure S1) were incorporated in a site-specific manner for short RNAs or were incorporated randomly during *in vitro* transcription of long RNA transcripts by utilizing a mixture of modified and unmodified UTP nucleotides. We use either biotin-containing or azide-containing ribonucleotide modifications, the latter subsequently post-transcriptionally labeled with biotin. We used a complete substitution with biotin-UTP in the case of modified short RNA transcripts, as the short RNA contains a single uridine site and a 1:10 azide-UTP:unmodified UTP ratio in the case of long RNA. We achieved signal enhancement of biotin-containing RNAs by binding monovalent streptavidin (the structure of monovalent streptavidin with one biotin binding site is shown in Figure 1b), which enables the distinction between unmodified and modified short RNAs, as shown in the nanopore events.

We used glass nanopores to detect a DNA nanostructure with short RNAs or a long RNA nanostructure by applying a voltage across the pore (Figure 1c). Negatively charged nucleic acid nanostructures translocate through the pore toward the positively charged electrode by an electrophoretic force.^19,40,41^ Once the duplex section of the nanostructure enters the nanopore, it partially blocks the ionic current, and when the section with a physical protrusion passes through the nanopore, it blocks the current further, leading to the downward current spike^16,31^ as seen in example events in panels d and e of Figure 1. In particular, the binding of monovalent streptavidin to the biotin-modified uridine produces a larger current drop in the ionic current when the nanostructure translocates through the nanopore.^16^ The variation in current drop in downward nanopore spikes between measurements is attributed to differences in pore diameter (Figure S15).^38^ We discriminated between unmodified and modified short RNAs (biotin-11-uridine modifications with monovalent streptavidin) by examining the magnitude of the current spike drop (Figure 1d). Unmodified RNA forms a duplex causing a smaller current downward spike (in gray), where modified RNA made with biotin-11-UTP once bound by monovalent streptavidin causes a larger current downward spike (in blue). Hence, we can discriminate the incorporation of a single biotin-11-modified UTP in short RNA. Similarly, the long RNA nanostructure with modifications along the RNA strand is mapped using nanopores, allowing us to determine the position and estimate the level of modifications by observing the downward current spike magnitude in the nanopore events (Figure 1e).

## Detecting Modified Nucleotide Incorporation in Short RNA Transcripts

We demonstrated the detection of modified short RNA by performing *in vitro* transcription with unmodified UTP and 5-modified UTP. As a template, we constructed an 80 bp DNA containing a T7 RNA polymerase promoter to yield a 63-nucleotide RNA (Figure 2a; oligonucleotide sequences are listed in Table S1). To generate short RNAs with a modified UTP, we used biotin-11-UTP in the reaction mixture where RNA has a single uridine site (Figure 2b; termed construct B in Table S1), and for the unmodified form, only unmodified UTP was added (Figure 2c; termed construct A in Table S1). As a result, the short RNAs either contain or lack biotin modifications. A polyacrylamide gel electrophoresis (PAGE) gel of the DNA templates and transcription products are shown in Figure S2. We then assembled a 2.7 kbp DNA nanostructure with two single-stranded DNA overhangs that specifically target each of these transcripts in a sequence-dependent manner (Figure 2d; design details are shown in Figure S3; the agarose gel of the assembly is shown in Figure S4; sequences are listed in Tables S2 and S3). Both RNAs were hybridized to the overhangs of the DNA nanostructure, which was then mixed with streptavidin (Figure 2e). The hybridization of the RNAs with the DNA overhangs and the subsequent labeling with streptavidin were further validated via PAGE (Figure S5). We achieved signal enhancement of biotin-modified RNA by binding streptavidin, which enabled the distinction between unmodified and modified short RNAs with nanopores. In the nanopore translocation events, two downward spikes are depicted (Figure 2f). The smaller downward spike is attributed to unmodified 63 nt RNA hybridized to its overhang (gray), while the deeper spike is attributed to modified 63 nt RNA with biotin bound to streptavidin (in blue); additional nanopore events are shown in Figure S6.

**Figure 2:**
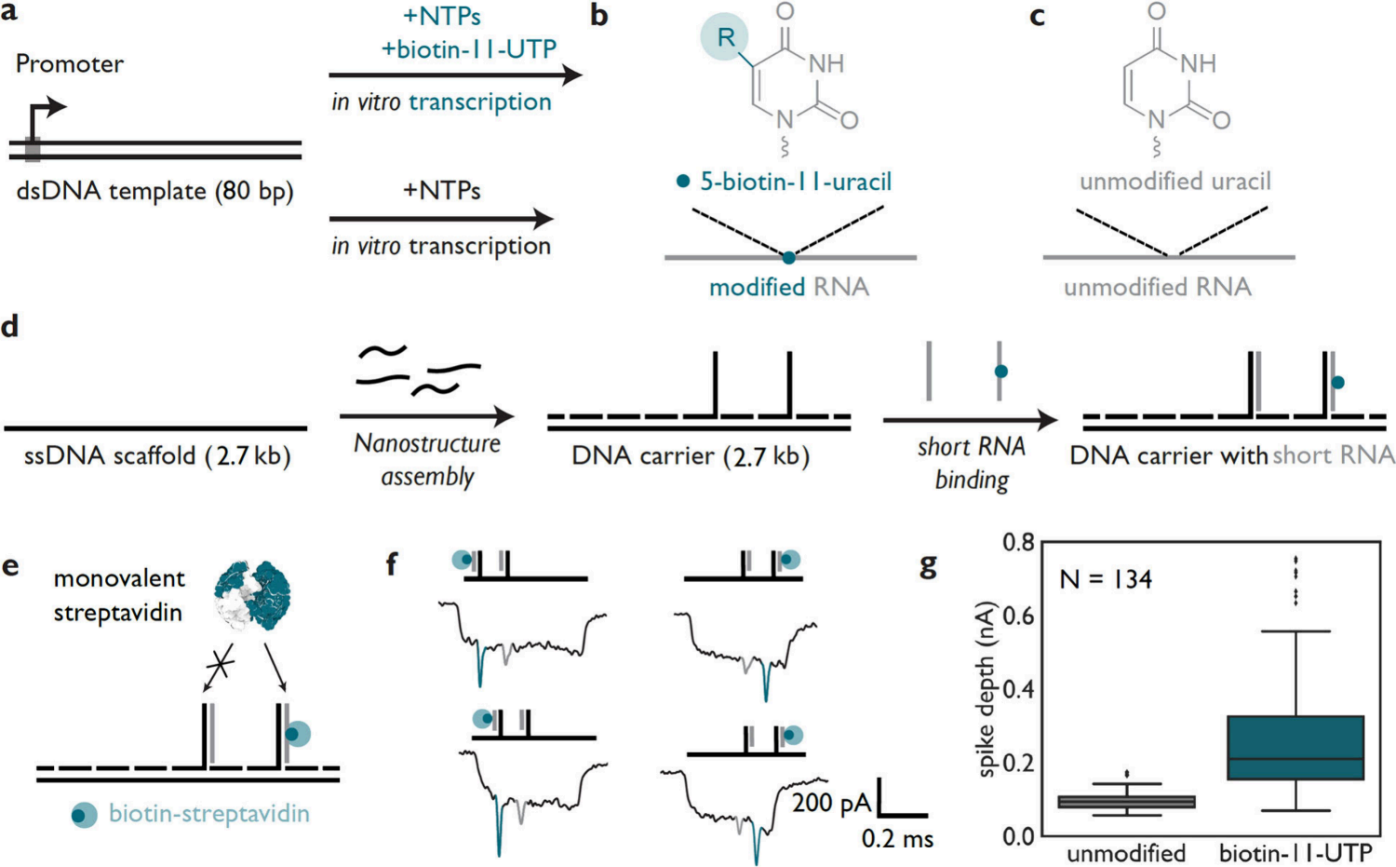
Nanopore detection of modified short RNAs with DNA nanostructure. (a) The DNA construct (80 bp) is *in vitro* transcribed using T7 RNA polymerase either using biotin-11-UTP or UTP, yielding modified or unmodified RNA, respectively. (b) The 5-carbon position of the uracil within UTP is colored blue, showing the biotin-11 modification incorporated into RNA. (c) *In vitro* transcription with no modified UTP yields unmodified RNA. Structure of unmodified uracil within UTP. (d) We assembled a DNA nanostructure by binding short oligonucleotides to a single-stranded DNA scaffold (2.7 kb). Then, short RNAs were bound to the resulting DNA nanostructure (2.7 kbp) through hybridization with the DNA overhangs. (e) Monovalent streptavidin binds to the biotin group of the modified RNA but does not bind to unmodified RNA. (f) We measured the DNA nanostructure bearing short RNAs with nanopores. The DNA nanostructure carrying short RNAs is translocated through a nanopore producing a characteristic ionic current readout. The nanopore events show two current spikes where the gray spike corresponds to the binding of unmodified RNA to the nanostructure, and the blue spike is ascribed to biotin-containing RNA. (g) The prominence of the current spike ascribed to the biotin-containing RNA is greater than for the unmodified RNA. Modified biotin-11-UTP RNA and unmodified RNA that have median values of 0.21 and 0.09 nA, respectively, are depicted.

The magnitude difference between current spikes is observed due to ion depletion during the translocation of streptavidin through the nanopore, making the blue current spike more prominent than the gray spike in the current trace. The current spike ascribed to modified RNA has a median depth of 0.21 nA, while the spike ascribed to the unmodified RNA has a median depth of 0.09 nA. The *p* value for a *t* test with unequal variance (Welch test) between both populations is 6.53 × 10^–22^. The change in the current spike depth enables the distinction between the modified and unmodified transcripts (Figure 2g). These modulations in ionic current demonstrate how site-specific labeling can be used in tandem with nanopore microscopy to detect short, modified RNA species.

## Template-Free, Catalyst-Free Click Chemistry between Nucleic Acids Is Affected by Salt Charge Screening and Linker Chemistry

We aimed to elucidate the conditions for efficient template-free, catalyst-free click chemistry between nucleic acids to enable the quantitative labeling of bioorthogonal RNA modifications. Azide and other click chemistry groups are attractive functional handles due to their size and inertness in biological systems, enabling their incorporation during *in vitro* transcription.^42^ To enable quantitative labeling, we screened conditions for efficient click chemistry between azide-containing nucleic acids and a dibenzocyclooctyne (DBCO) oligonucleotide (Figure 3). Specifically, we assessed the role played by nucleic acid structure, the modification’s linker chemistry, and the salt concentration in influencing the reaction yield (Figure 3).

**Figure 3:**
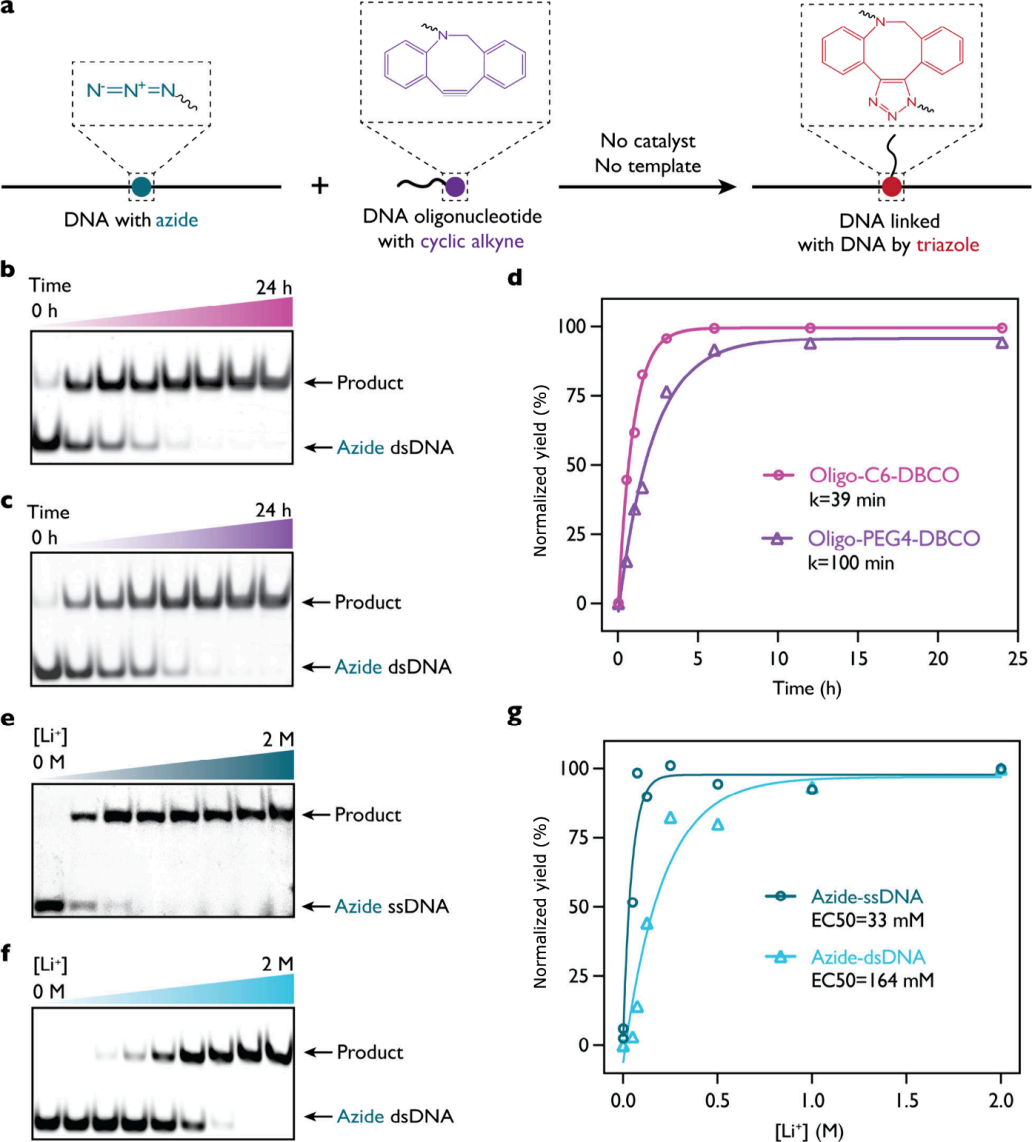
Template-free, catalyst-free click chemistry is influenced by the nucleic acid structure and linker chemistry. (a) An internally positioned azide modification in a single-stranded (ss) or double-stranded (ds) DNA context (26 nt or 26 bp) was incubated with 3′-DBCO DNA oligonucleotide to yield a triazole three-way branched nucleic acid construct. We assessed the influence of the linker chemistry between 3′-DBCO and oligonucleotide with (b) a six-carbon linker (C6) and (c) a polyethylene glycol (PEG4) linker on the labeling of azide-modified dsDNA in 2 M LiCl. (d) The reaction half-times for the C6 and PEG4 linkers are 39 and 100 min, respectively, showing that the C6 linker enables the reaction to reach a plateau within 6 h in a double-stranded context. The effect of concentration of LiCl on the reaction yield, after incubation for 1 h at 37 °C, between a 15 nt 3′-C6-DBCO oligonucleotide and azide modification is shown for (e) ssDNA and (f) dsDNA contexts, where the 3′-C6-DBCO oligonucleotide is in 7.5 times molar excess compared to the azide. (e) The DNA oligonucleotide with a 3′-terminal DBCO reacts with an ssDNA containing an internal azide modification. The product represents the covalent triazole branched DNA structure. (f) Similarly, the reaction under the same conditions with dsDNA containing an internal azide modification necessitates higher LiCl concentrations for the reaction to reach completion. (g) The half-maximal effective concentrations of LiCl (EC_50_) for the reaction with azide modification in ssDNA and dsDNA contexts are 33 and 164 mM, respectively, where we observe that the dsDNA azide has an order of magnitude higher triazole formation rate constant than in an ssDNA context.

We delved into the impact of linker chemistry on the efficiency of the click reaction by comparing the use of a carbon 6 (C6) linker versus a poly­(ethylene glycol) (PEG4) linker in the DBCO-conjugated oligonucleotide (Figure 3b–d). These two linkers were selected due to their distinct physical properties, with C6 serving as a short, rigid alkyl chain and PEG4 representing a longer, flexible polymer, thus providing a representative contrast in linker flexibility and steric behavior. Our results show that the reactions utilizing the C6 linker reached half-completion in 39 min, whereas those with the PEG4 linker took significantly longer, with a half-time of 100 min (Figure 3d). This indicates that the C6 linker enhances the reaction’s efficiency, allowing it to nearly reach completion within a 6 h time frame. The shorter C6 linker may offer improved DBCO exposure compared to the longer PEG4, which is prone to coiling in high monovalent ion concentrations.^43^ This compact conformation likely restricts DBCO accessibility, thereby reducing its reactivity with azide-modified DNA. While alternative linker architectures may further improve reaction kinetics and shorten labeling times, the C6 linker achieves nearly complete conjugation within 6 h, underscoring its functional effectiveness.

Then, we explored how the concentrations of monovalent salts, including LiCl (NaCl and KCl data are shown in Figures S7 and S8), impact the yield of click chemistry reactions involving a 15 nt oligonucleotide with 3′-C6-DBCO oligonucleotide and azide-modified single-stranded DNA (ssDNA; 26 nt azide modification positioned internally) and double-stranded DNA (dsDNA; 26 bp) (Figure 3e–g; oligonucleotides are listed in Table S4). In the experiments focused on ssDNA, the oligonucleotide featuring 3′-C6-DBCO was incubated with ssDNA with an internal azide modification (Figure 3e). This design resulted in the formation of a covalent triazole three-way branched DNA structure, demonstrating the effectiveness of the click chemistry reaction in facilitating the linkage between the modified ssDNA and the DBCO oligonucleotide. However, we do not observe a significant difference in yield when testing different concentrations of DBCO oligonucleotides or different buffer pHs when supplied with monovalent ions, the reaction reaching completion under all conditions (Figures S9 and S10, respectively).

Conversely, when the click chemistry reaction was performed with 26 bp dsDNA containing an internal azide group (Figure 3f) and the 3′-C6-DBCO oligonucleotide, it was observed that a LiCl concentration of >1 M was required to achieve completion. This indicates that the structural rigidity and the spatial arrangement of dsDNA necessitate enhanced reaction conditions to facilitate the efficient linkage, highlighting the importance of considering the physical and chemical environment of the nucleic acids. The half-maximal effective concentration (EC_50_) of LiCl for the reaction to proceed in ssDNA and dsDNA contexts increased up to 5-fold from 33 to 164 mM (Figure 3g). The difference in the product yield with dsDNA and ssDNA underscores the more challenging nature of click chemistry reactions involving dsDNA, which requires a higher salt concentration than those with ssDNA, reflecting the structural considerations that must be taken into account. These mechanistic insights from DNA-based labeling reactions informed the design of our RNA labeling with a 3′-C6-DBCO oligonucleotide. We note that the efficiency of catalyst-free click chemistry here is primarily governed by electrostatic repulsion between negatively charged nucleic acid backbones. Hence, we are expecting that these conditions can be readily applied to RNA.

These findings collectively highlight the complex interplay among the nucleic acid structure, linker chemistry, and salt concentration in determining the yield of template-free, catalyst-free click chemistry for nucleic acid modifications. Understanding these parameters is crucial for optimizing the methodology for quantitative labeling of nucleic acids.

## RNA Nanostructures Enable Relative Quantification of Cotranscriptionally Incorporated Modified Nucleotides in Long RNA

We utilized the 3′-C6-DBCO oligonucleotides for the labeling of long, modified RNAs, as depicted in Figure 4. A DNA template (3 kbp; the design of the plasmid and its sequence are in Figure S11 and Table S5) harboring a T7 RNA polymerase promoter served as the template for *in vitro* transcription, producing a 3094 nt RNA scaffold (Figure 4a; sequence shown in Table S6). Azide-C3-UTP nucleotides were included into the nucleotide mix during *in vitro* transcription, facilitating their cotranscriptional incorporation within the RNA scaffold. We selected a partial UTP substitution (1:10 ratio of azide UTP:UTP) that maintains efficient T7 polymerase activity and high yield of full length transcripts,^44^ while providing sufficient modification levels per molecule. DBCO-oligonucleotides are covalently linked, under a molar concentration of LiCl, with azide-modified RNA nucleotides. Leveraging the DBCO-labeled RNA, we then assembled an RNA nanostructure using the 3′-biotinylated oligonucleotide to enhance the structural signal and short DNA oligonucleotides to form an RNA:DNA hybrid (Figure 4b; oligonucleotides are listed in Table S7). Nanopore microscopy identifies the downward spikes on the RNA nanostructure and thus their relative positions and levels of the underlying modifications (Figure 4c). Streptavidin conjugation to biotin significantly amplifies nanopore signal intensity compared to signal produced from oligonucleotide overhangs, enabling more robust detection through enhanced spike prominence.^45^ This is evident from the fluctuating spike count and depth in nanopore events, as showcased in panels d and e of Figure 4 (additional events are shown in Figure S12, and a scatter plot of the mean event current versus event duration is shown in Figure S13a). The normalized positions of the labels, visible as downward spikes in the nanopore events, are plotted versus the whole nanopore event duration (Figure 4f), observed as an even distribution across the length of the molecule, consistent with the random incorporation pattern of the modified nucleotides during transcription. When the distance between modifications is less than 150 bp, their current downward spikes are shown as a single, compound peak with a deeper current drop.^16^

**Figure 4:**
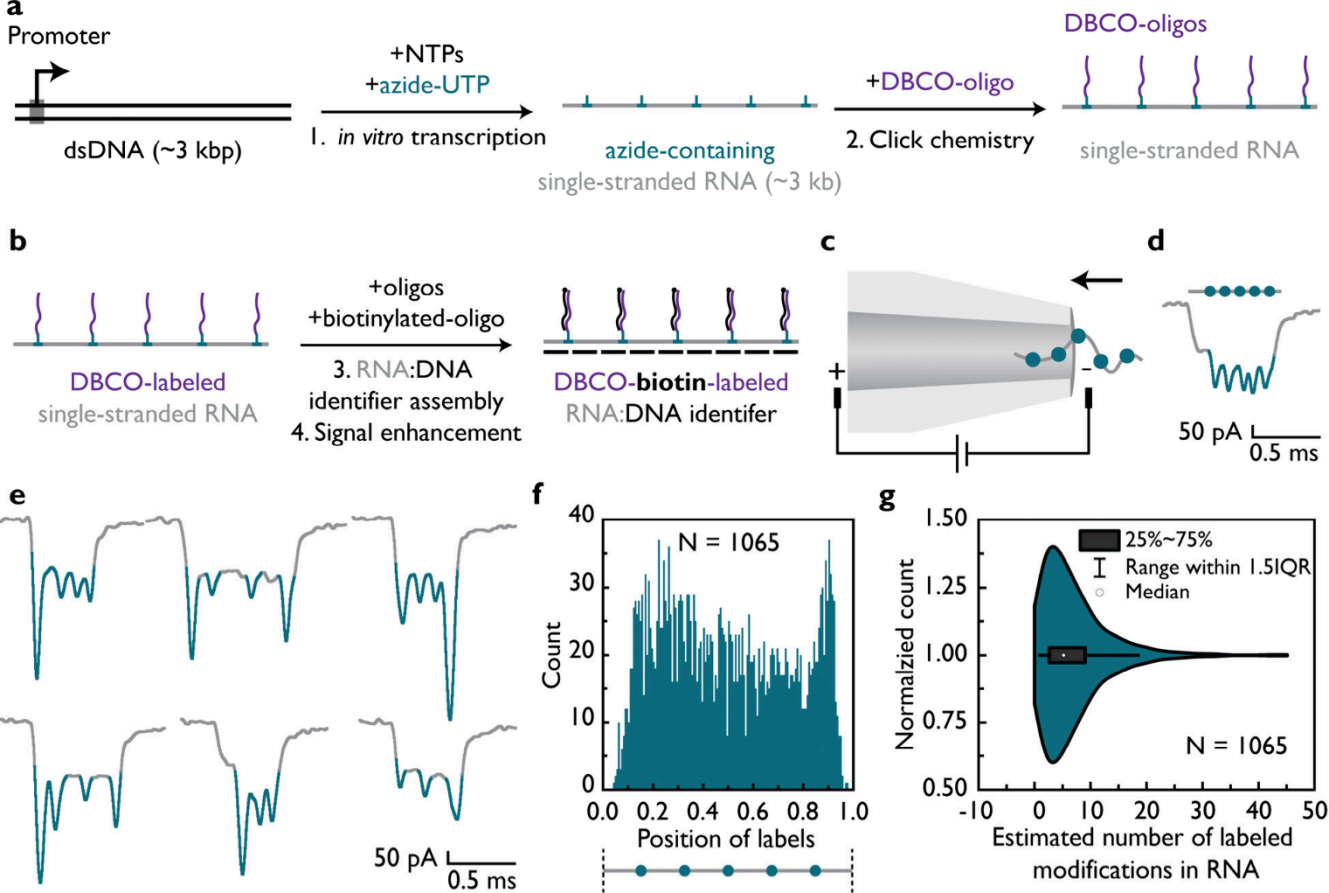
Nanopore detection of cotranscriptionally labeled long modified RNA. (a) The DNA template (3 kbp) containing a T7 RNA polymerase promoter was used for *in vitro* transcription. Azide-containing UTP (blue) was used to produce a modified RNA scaffold. Labeling with a 3′-C6-DBCO oligonucleotide (purple) proceeds through catalyst-free click chemistry covalently attached to the oligonucleotides to the RNA. (b) DBCO-labeled modified RNA was used as a scaffold to assemble the RNA nanostructure. The 3′-biotinylated oligonucleotide facilitates signal enhancement though its binding to streptavidin. (c) A nanopore microscope enables the detection of modified nucleotides along the RNA nanostructure as shown by the variable spike number and depth in nanopore events in panels d and e depicting the incorporation level of azide-labeled UTPs. (f) Histogram of the downward spike positions along RNA indicating the distribution of modified uridines in RNA. (g) We estimated the number of labeled modifications per RNA calculated from the spikes’ current drop. The violin plot shows the average number of labeled modifications as being 7.2 ± 8.1 per RNA molecule. The sample size included 1065 nanopore events.

Leveraging the peak area or current drop amplitude, these signals can be attributed to a specific number of closely spaced labeled modifications. A larger population of modifications is observed toward the ends of the molecules attributed to 3′ truncations of T7 RNA polymerase^46^ and partial folding of molecules.^36^ To estimate the number of modifications per transcript, we measured the total ionic current blockade depth for each spike, which could correspond to one or more streptavidin-labeled modifications. These values were then normalized to the magnitude of a single streptavidin to provide an estimated number of streptavidin molecules for each spike,^16,23^ allowing us to estimate modification counts (Figure 4g; the estimated number vs spike position is shown in Figure S13b). We found that on average, there are 7.2 ± 8.1 labels per RNA molecule.

In addition, we applied the labeling technique to a dsDNA nanostructure, where the azide modifications were in predefined positions, and showed that both 3′-terminal and internal azide modifications are labeled in long nucleic acid molecules (Supplementary Section I and Figure S14). Our results underscore our capability to identify modifications of long RNA or DNA with nanopores by employing cotranscriptional incorporation of modified nucleotides.

We developed a solid-state nanopore-based approach utilizing RNA and DNA nanostructures to detect cotranscriptionally incorporated modifications in RNA. We demonstrated the detection of incorporated biotin-modified uridine in short RNA and azide-modified uridine in long RNA, establishing a solid-state nanopore sensing strategy for single-molecule detection of incorporated modified nucleotides in RNA from tens of nucleotides to kilobases in length.

Metabolic labeling studies require methods that can provide relative quantitative insights into the number of nascent transcripts and the broad spatial distribution of nucleotide modifications. It is important to point out that the single-transcript level base pair resolution is not always necessary.^4,7^ Our study demonstrates the feasibility of detecting biotin- and click-labeled short and long RNAs using *in vitro*-transcribed RNA. While promising, future studies will be necessary to validate this strategy for the detection of both short and long cellular RNA. A potential future application of this method is the transcript-specific temporal tracking, as our platform can detect relative differences in modification levels and their broad spatial distribution along *in vitro*-transcribed RNAs.^16,33^

Existing methods for detecting modified uridine-containing short RNAs face challenges due to the transcripts’ small size, which limits their efficient enrichment and sequencing.^7,47^ Traditional approaches, such as those relying on affinity purification with biotin, often introduce biases or fail to provide direct quantification of modified RNA incorporation since the labeling level is not retrieved after pull-down.^4^ As an alternative here, we enabled the detection of modified nucleotides and a semiquantitative approach to access the extent of modifications in long transcripts. Detecting the relative level of modified nucleotide presence might be more relevant for observing RNA age and tracking RNA processing.^9,11^ An additional advantage of our strategy is direct readout without the need for amplification or enzymatic conversion, reducing biases associated with sequencing methods.^15,48,49^

Current cotranscriptional labeling detection methods for long RNA transcripts require chemical labeling that introduces biotin for pull-down with streptavidin beads, followed by additional processing steps such as reverse transcription and sequencing, which introduce bias and misrepresent the transcripts’ relative abundance.^11,12,18,47^ In particular, pull-down approaches cannot accurately track transcript histories due to a lack of quantitative information.^5,7,9^ Our method provides an alternative by leveraging RNA:DNA nanostructure-assisted nanopore sensing to detect cotranscriptionally introduced modified nucleotides in long RNA. We found conditions for the efficient post-transcriptional labeling of azide-modified uridine in long RNA with DNA-cyclooctyne labels.

Click chemistry has emerged as a powerful tool for bioorthogonal labeling, particularly in RNA metabolic studies where azide-modified nucleotides serve as attractive click handles for *in vivo* applications.^11,42,50–52^ Prior studies have demonstrated the utility of click reactions for RNA enrichment and detection.^11^ Leveraging the unique nanopore signatures of RNA molecules, this approach allows for the detection of modified nucleotides in both short and long RNAs through nanostructure-enabled sensing.

Our study establishes a nanopore-based framework for detecting modified nucleotide incorporation in RNA, expanding existing approaches for the detection of modified nucleotides in RNA transcripts of varying lengths. By leveraging DNA and RNA nanotechnology and nanopore sensing, we provide a versatile platform for studying modified nucleotide incorporation into RNA at the single-molecule level. This method offers new avenues for the exploration of post-transcriptional *in vivo* RNA metabolic labeling and therapeutic RNA metabolism.

## Supplementary Material



## Notes

Supplementary text, materials and methods, including Figures S1–S15 and Tables S1–S9 (PDF)
