# Supplementary material for: Programmable RNA Nanostructures Enable Nanopore Detection of Cotranscriptionally Introduced RNA Modifications
Source: Nano Lett. 2025 Aug 4;25(32):12184–92. doi: 10.1021/acs.nanolett.5c02391 (PMC12356122; doi:10.1021/acs.nanolett.5c02391)
Supplement: Supplementary file 1 [file nl5c02391_si_001.pdf]

Supporting Materials for

Programmable RNA nanostructures enable nanopore detection of co-transcriptionally introduced RNA modifications

Iva Mohora‡[1], Gerardo Patiño Guillén‡[1], Kevin Neis[2], Julián Valero[2,3], Ulrich F. Keyser*[1], Filip Bošković*[1]

[1]Cavendish Laboratory, University of Cambridge, 19 JJ Thomson Avenue, Cambridge CB3 0HE, United Kingdom

[2] Interdisciplinary Nanoscience Center (iNANO), Aarhus University, DK-8000, Aarhus, Denmark

[3] Department of Molecular Biology and Genetics, Aarhus University, DK-8000, Aarhus, Denmark

‡Authors contributed equally

*E-mail: fnb24@cam.ac.uk, ufk20@cam.ac.uk

This PDF file includes

Materials and Methods
Supplementary Text
Figs. S1-S15
Tables S1-S9

Supplementary Materials and Methods

Materials

The commercial buffers used in this study were Tris-EDTA buffer solution 100 × concentrate (Sigma-Aldrich, catalog number T9285), 0.2 µm filtered 1 M $MgCl_2$ (Invitrogen by Thermo Fisher Scientific, catalog number AM9530G), 0.2 µm filtered and autoclaved nuclease-free water (Ambion, catalog number AM9937). Lithium chloride for molecular biology ≥99% purity (Sigma-Aldrich, catalog number L9650), sodium chloride for molecular biology ≥99% purity (Sigma-Aldrich, catalog number S3014), Tris HCl BioPerformance certified, ≥ 99% purity (Sigma-Aldrich, catalog number T5941). All buffers used in this study were filtered with 0.22 µm Millipore syringe filter units (MF-Merck Millipore™, catalog number GSWP04700).

Glass quartz capillaries with filament (inner diameter 0.2 mm, outer diameter 0.5 mm) were purchased from Sutter Instrument US. PDMS was purchased from Sylgard 184, Dow Corning (101697), microscope slides clear ground 1.0 – 1.2 mm (Thermo Fisher Scientific, catalog number 1238-3118), silver wire with 1.0 mm diameter (Advent Research Materials Ltd, catalog number AG548711). Amicon 0.5 mL filter units (100 kDa cut-off) were purchased from Merck (catalog number UFC5100BK).

DNA LoBind® Tubes 0.5 mL and 1.5 mL (Eppendorf) were purchased from Thermo Fisher Scientific, and thin-walled, frosted lid, RNase-free PCR tubes (0.2 mL) were purchased from Thermo Fisher Scientific (catalog number AM12225). Single-stranded circular m13mp18 7,249 nt in length was purchased from Guild Biosciences or NEB.

The modified UTPs were purchased from Jena Biosciences, Germany namely biotin-11-UTP (NU-821-BIOX), and HighYield T7 Azide RNA Labeling Kit with 5-Azido-C3-UTP (RNT-101-AZ). T7 RNA polymerase kit for *in vitro* transcription with biotin-11-UTP bought from NEB (E2040S). All restriction enzymes were purchased from NEB namely, EcoRI-HF (R3101T), BamHI-HF (R3136T), DraIII-HF (R3510S), and AfeI (R0652S). DNA purification after enzyme digestion was performed using Monarch® PCR & DNA Cleanup Kit (5 µg) from NEB. All individual oligonucleotides were purchased from

Integrated DNA Technologies (IDT) at 100 nmol scale with standard desalting. Oligonucleotides longer than 60 nt were subjected to PAGE purification (IDT) and azide-containing oligonucleotides were HPLC purified (IDT). DBCO-containing oligonucleotides were purchased from Eurogentec or Biomers with HPLC purification. RNA purification after *in vitro* transcription was performed with the Monarch® RNA Cleanup Kit (10 μg or 50 μg).

METHODS

In vitro transcription of short RNAs

In vitro transcription for the production of short RNAs was performed with an 80 bp double stranded DNA template containing a T7 promoter, yielding 63 nt long transcripts. Preparation of the DNA templates was done by annealing single stranded DNA oligonucleotides (sequences in **Table S1**) at 90 °C for 2 minutes, 65 °C for 5 minutes and 37 °C for 5 minutes.

The transcription reactions were performed using a Y639F mutant T7 RNA polymerase (T7RNAP; Aptamist, Denmark). We used 2 mM ATP, CTP and GTP, and 2 mM UTP or biotin-11-UTP to produce unmodified and modified transcripts, respectively. The reaction volume was 10 μL and it included 80 mM HEPES (pH 7.5), 25 mM $MgCl_2$, 2 mM spermidine-HCl, 1 U/μL SUPERase·In RNase Inhibitor (AM2696, Invitrogen), 50 μg/mL bovine serum albumin, 0.005 U/μL inorganic pyrophosphatase, 10 mM dithiothreitol.

DNA nanostructure assembly for capture of short RNAs

The assembly of DNA nanostructures to study modifications in short RNA was done by mixing 2.7 kb single stranded DNA (2,680 nt fragment of M13mp18). The single stranded DNA fragment was produced by restriction digestion of single-stranded M13 DNA (M13mp18, 7,249 nt, 100 nM, Guild Biosciences with DraIII-HF (New England Biolabs (NEB), R3510S) and AfeI (NEB, R0652S) in 1 × rCutSmart Buffer (NEB) following a protocol reported previously. (*1*, *2*)

DNA was purified using Monarch PCR & DNA Cleanup Kit (5 μg) (NEB, T1030S) following the manufacturer's instructions. Then, the DNA nanostructure was assembled by

mixing the single stranded DNA with the DNA oligonucleotides found in **Table S3** and in the presence of the targeted transcripts. The 40 µl reaction included 800 fmol of single stranded DNA scaffold (20 nM final concentration), 2400 fmol of complementary oligonucleotides (60 nM final concentration), and 7,200 fmol of RNA targets (180 nM final concentration). The mixture was prepared in 10 mM $MgCl_2$, 10 mM Tris HCl buffer (pH 8.0), and nuclease-free water was added to reach the final reaction volume. Before use, nuclease-free water was filtered using MF-Millipore membrane filters (0.22 µm pore size) and irradiated with UV light for 10 minutes. The reaction components were mixed by pipetting and briefly spun down. The assembly was done by heating to 70 °C for 30 seconds and gradually cooling to room temperature over 45 minutes (90 cycles of 30 seconds with a 0.5 °C decrease per cycle). RNA IDs were filtered twice using 0.5 mL 100 kDa cut-off Amicon filters to remove excess oligonucleotides. 10 mM Tris HCl pH 8.0 with 0.5 mM $MgCl_2$ was used as a washing buffer for filtration.

Electrophoretic mobility shift assay

Electrophoretic mobility shift assay (EMSA) was used for the characterization of the DNA nanostructures with1 % (w/v) agarose gel, 1 × TBE, 0.02% sodium hypochlorite. 100 to 200 ng of each sample was added per lane, and the assay was run for 3 hours with an applied voltage of 80 V.

Reaction between azide-modified and DBCO-modified oligonucleotides

We mixed a 15 nt DBCO-oligonucleotide with a 26 nt azide-oligonucleotide (**Table S4**) in the molar ratio of 1:1, respectively, in 0-2 M (0, 0.05, 0.075, 0.125, 0.25, 0.5, 1, 2 M) monovalent salt at room temperature for 1 h.

Reaction between DBCO-modified oligonucleotide and azide-modified duplex

Azide-oligonucleotide and its complementary oligonucleotide (**Table S4**) were mixed to a 10 µM final concentration, in a molar ratio of 1:1, in the desired salt concentration and pH. The mixture was heated for 5 min at 70 °C and then slowly cooled down to 25 °C over 40 min to facilitate annealing. We mixed the DBCO-oligonucleotide with 26 bp azide-dsDNA

in the molar ratio of 7.5:1 respectively in 2 M LiCl at room temperature or 37 °C for 1 h unless otherwise indicated.

In vitro transcription of long RNA with azide modified nucleotides

We propagated plasmid DNA (**Figure S10**; **Table S5**) in *Escherichia coli* DH5alpha as previously described. (*3*) The plasmid was linearized using DraIII-HF (NEB) for 2 h at 37 °C and purified with Monarch PCR and DNA clean up kit (NEB). The concentration was quantified with a Nanodrop UV spectrometer. Linearized DNA was used as a template for *in vitro* transcription with high-yield T7 RNA polymerase (NEB) and by adding 8.75 mol% of azide-C3-UTP (Jena Biosciences). The 20 µl reaction included a final concentration of 1 mM of ATP/GTP/CTP mix, 0.65 mM unlabeled UTP, 0.35 mM azide-C3-UTP, 1 µg of template DNA and 10 mM dithiothreitol. The reaction was incubated for 4 hours at 37 °C. The mix was treated with DNaseI (NEB) for 15 min, 37 °C and subsequently purified with Monarch RNA purification kit (NEB). The concentration was quantified with a Nanodrop UV spectrometer and stored in -80 °C freezer until use.

Reaction with *in vitro* transcribed modified RNA

The purified RNA was reacted with the DBCO-C6 oligonucleotide (Table S4) in a 8 µl reaction in a final concentration of 2 M LiCl, 1 µM DBCO-C6 oligonucleotide and a final concentration of 10 nM of the RNA. The reaction was incubated at 37 °C for 14 hours.

RNA nanostructure assembly

We prepared a 40 µl reaction by mixing *in vitro* transcribed RNA (to 20 nM or 800 fmol) and oligonucleotides (to 60 nM each or 2,400 fmol) in 100 mM LiCl, 1× TE buffer (10 mM Tris-HCl buffer, 1 mM ethylenediaminetetraacetic acid, pH 8.0) and nuclease-free water was added to the final reaction volume. Buffers were filtered with the MF-Millipore membrane filter with 0.22 µm pore size. The reaction was mixed by pipetting and spun down. The mixture was heated to 70 °C for 30 s and then gradually cooled (−0.5 °C per cycle, 90 cycles of 30 s each) over 45 minutes to 25 °C and held at 4 °C. RNA nanostructure was filtered using 0.5 ml 100 kDa cut-off Amicon filter units. The washing buffer used for filtration was composed of filtered 10 mM Tris HCl pH 8.0 with 0.5 mM $MgCl_2$.

Native polyacrylamide gel electrophoresis (PAGE)

The gels were run in a Mini-PROTEAN Tetra Cell (Bio-Rad). Gels were hand cast at a final concentration of 10% (v/v) polyacrylamide with 1 × Tris–borate–EDTA (TBE) pH 8.0. Per 15 ml of gel mixture, 150 µl 10% (w/v) ammonium persulfate solution (aliquots kept at −20 °C and freshly thawed) and 10 µl *N*,*N*,*N′*,*N′*-tetramethylethylenediamine (TEMED) were added to start the polymerization which was left to set in gel plates (Bio-Rad, catalog number 1653311) for at least 1 h before running. Samples were run for 80 min, in 1 × TBE buffer, at 80 V, in an ice bath. We loaded 1 µl of 10 µM samples or otherwise if indicated, mixed with 1 µL of 6 × TriTrack DNA Loading Dye (ThermoFisher Scientific, catalogue number R1161). GeneRuler Ultra Low Range DNA Ladder (ThermoFisher Scientific, catalogue number SM1213) was loaded into the first or final well. The gel was poststained in 3 × GelRed buffer (Biotium, catalog number 41001) for 10-15 min and imaged with GelDoc- It^{TM} (UVP). Gel images were processed using Fiji ImageJ software by inverting greyscale, homogenous background subtracted with 300 pixels rolling ball radius and removal of noise with despeckling tool and dark outlier removal at 50 threshold and 7-pixel radius and brightness and contrast adjusted. All adjustments were applied homogeneously to the entire image.

Long DNA nanostructure assembly with azide modifications

We prepared ssDNA scaffold by linearizing single-stranded M13 DNA (M13mp18, 7,249 nt, 100 nM, Guild Biosciences). The DNA nanostructure, which consists of linearized M13 DNA (7,228 nucleotides long) fully hybridized by oligonucleotides, forms a nicked double-stranded structure with two four-thymidine overhangs at each end to prevent multimer formation. The mixture included 20 nM of M13 DNA, 60 nM of oligonucleotides (threefold excess over M13 DNA; **Table S8** and **Table S9**), 10 mM $MgCl_2$, and 1× TE buffer (10 mM Tris HCl, 1 mM EDTA, pH 8.0). This mixture was mixed by pipetting, centrifuged, and then heated to 70 °C for 30 seconds followed by a 45-minute cooldown to 25 °C and held at 4 °C. Excess oligonucleotides were removed with Amicon Ultra 0.5 mL centrifugal filters with a 100 kDa cutoff, using a wash buffer composed of 10 mM Tris-HCl (pH 8.0) and 0.5 mM $MgCl_2$. 40 µL of the reaction mixture was added to 460 µL of 10mM Tris-HCl (pH 8.0), 0.5

mM $MgCl_2$ and centrifuged at 9200 × g for 10 minutes at 4 °C. 460 µL more 10 mM Tris-HCl (pH 8.0), 0.5 mM $MgCl_2$ was added, and the sample centrifuged again for 10 minutes, at 9200 × g and 4 °C. The sample was then recovered by turning the filter upside down and centrifuging for 2 min at 1000 × g. This yielded around 20-40 µL of the purified structure.

Reaction with DNA nanostructure

Oligonucleotides at specific positions along the DNA nanostructure contained azid modifications, namely at position 97, 121 and 142, either internally or at the 3′ end (**Table S9**). The mixture was incubated over week at 37 °C in a 1:7.5 molar ratio. The reaction outlined above was mixed in excess with a 15 nt biotin oligonucleotide (5`-/5bioTEG/TCACTCAGTATGGGT-3`) to facilitate streptavidin attachment.

Nanopore fabrication

To create nanopores with a size range of 10-15 nm, we employed a laser-assisted capillary puller (P2000F, Sutter Instruments). Capillaries made of glass with an outer diameter of 0.5 mm and an inner diameter of 0.2 mm, including a filament, were obtained from Sutter Instruments. The heating protocol utilized the following parameters: HEAT (470-500), VEL (25), DEL (170), and PUL (200).

Nanopore measurements

Nanopore measurements were conducted using the Axopatch 200B system in a solution containing 4 M LiCl, 1× TE buffer, pH 9.4. A constant voltage of 600 mV was applied during data collection. To identify individual events in the ionic current recordings, custom LabVIEW codes were employed. These codes utilized threshold parameters such as event duration, current drop, and event charge deficit. Background noise was effectively removed by referencing the mean event current of the double-stranded DNA:DNA duplex or double-stranded RNA:DNA duplex level, which allowed discrimination between duplex-level events and undesired molecules. Furthermore, event selection was refined by specifying ranges for event duration and event charge deficit, ensuring that only DNA and RNA duplex events were included while excluding aggregates and fragments. Specifically, unfolded (linear) DNA and RNA events were chosen for further analysis.

Supplementary section I

Short nucleic acid azide modification identification with DNA nanostructure

We show that covalent DNA labelling of long double-stranded DNA and single-stranded RNA facilitates their nanopore mapping and further expands the toolkit of DNA and RNA origami technology. We demonstrate the efficiency of covalently and site-specifically labeling a DNA nanostructure. A linearized single-stranded DNA derived from the M13 phage, comprising 7,228 nucleotides, served as a foundational scaffold onto which complementary oligonucleotides were attached (**Figure S14a**). Notably, within this DNA structure, three distinct sites were marked using azide labeled oligonucleotides, either internally or at the 3′-end, which are visually represented in blue. An intriguing aspect of this labeling process is the ability of the 20 nucleotide-long DBCO-oligonucleotide to specifically engage only with the azide modifications. This interaction gives rise to a stable triazole bond via a catalyst-free click chemistry reaction mechanism. Progressing further into the procedure, the triazole-labeled DNA nanostructure underwent hybridization with 3′-biotinylated oligonucleotides (**Figure S14b**). This particular step was pivotal in introducing a unique structural signature that could be visualized using nanopore microscopy (**Figure S14c**). With the aid of the glass nanopore microscope, the positions of labels along DNA nanostructure were successfully identified (**Figure S14d**). A representative microscopy event (**Figure S14d**) revealed three downward spikes, each of these spikes corresponding to one of the previously labeled sites on the DNA strand. Additional nanopore events are shown in **Figure S14e**.

References

1. F. Bošković, J. Zhu, R. Tivony, A. Ohmann, K. Chen, M. F. Alawami, M. Đorđević, N. Ermann, J. Pereira-Dias, M. Fairhead, M. Howarth, S. Baker, U. F. Keyser, Simultaneous identification of viruses and viral variants with programmable DNA nanobait. *Nat Nanotechnol* 18, 290–298 (2023).
2. N. A. W. Bell, U. F. Keyser, Digitally encoded DNA nanostructures for multiplexed, single-molecule protein sensing with nanopores. *Nat Nanotechnol* 11, 645–651 (2016).
3. G. Patiño-Guillén, J. Pešović, M. Panić, D. Savić-Pavićević, F. Bošković, U. F. Keyser, Single-molecule RNA sizing enables quantitative analysis of alternative transcription termination. *Nat Commun* 15, 1699 (2024).

Figure S1.

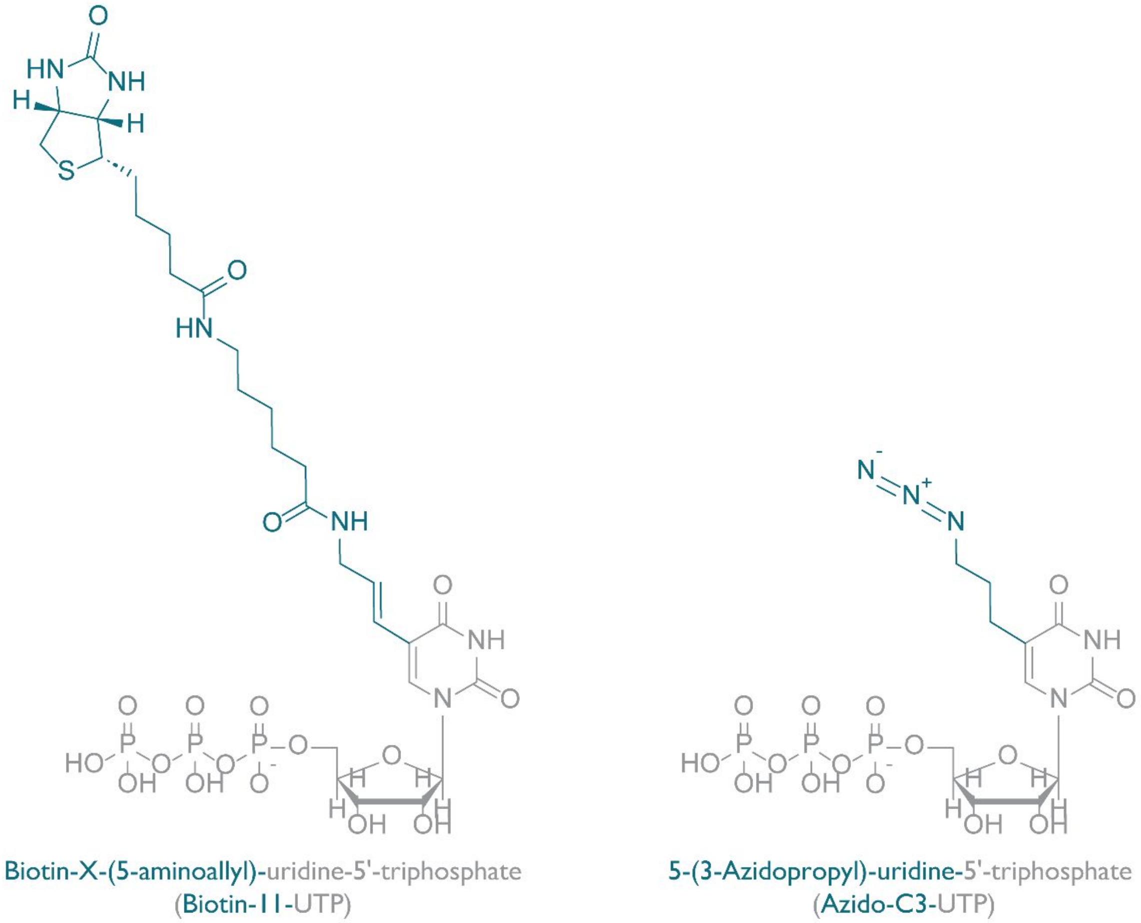

Figure S1. The detailed structure of modified UTPs used in this study for *in vitro* transcription reactions including biotin-11-UTP and azido-C3-UTP.

Figure S2.

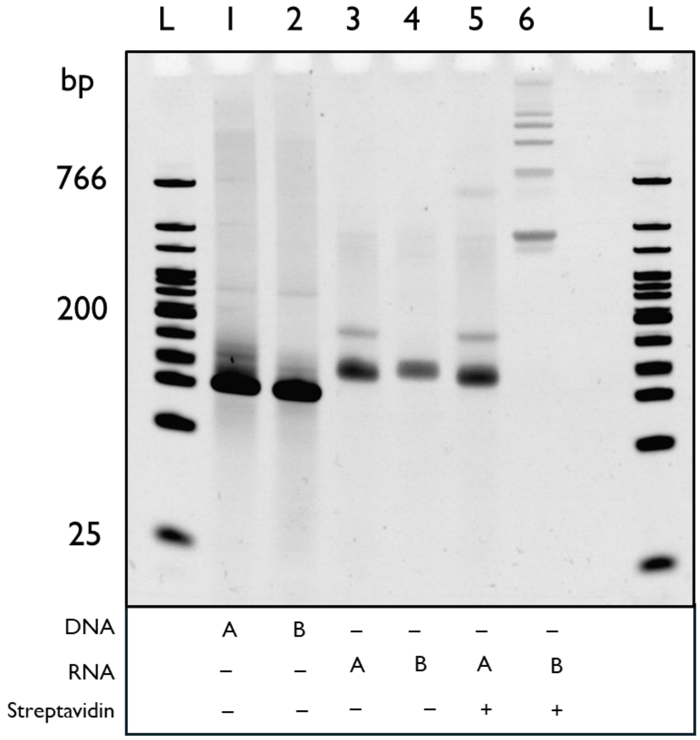

Figure S2. Incorporation of biotin UTP during transcription. The characterization of the assay was done *via* gel electrophoresis in a 10% (v/v) native polyacrylamide gel in 1 × TBE. **L** – Low molecular weight ladder. **1** – DNA template *A* (80 bp) **2** – DNA template *B* (80 bp). Both DNA templates contain a T7 promoter. Sequence in Table S1. **3** – RNA transcript from template *A*, does not contain biotin-11-UTP. **4** – RNA transcript from template *B* contains biotin on 5-position of uridine UTP. **5** – RNA transcript from template *A* with monovalent streptavidin added. **6** – RNA transcript from template *B* with monovalent streptavidin added.

Figure S3.

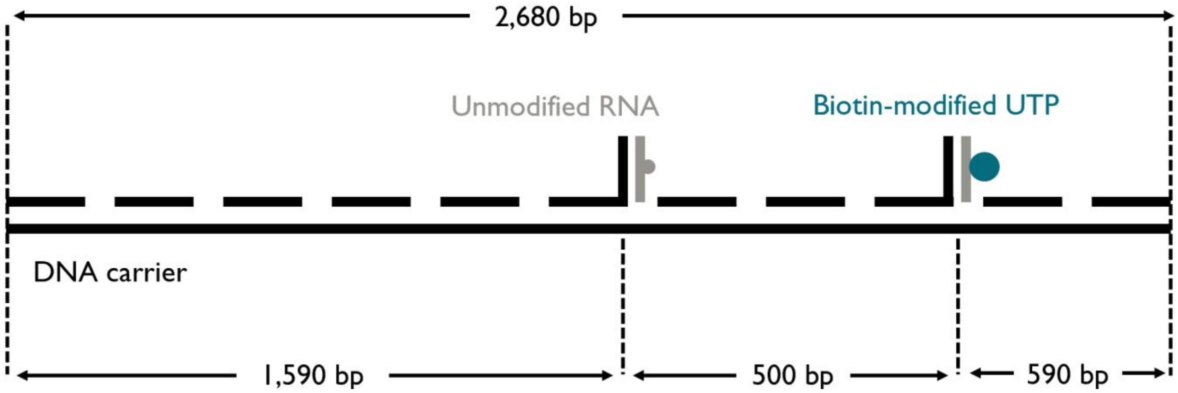

Figure S3. Design of DNA carrier with short RNA targets. The positions of the overhangs which capture the modified and unmodified short RNAs are shown. The sequence of the ssDNA backbone and the complementary oligonucleotides are found in Tables S2 and S3, respectively.

Figure S4.

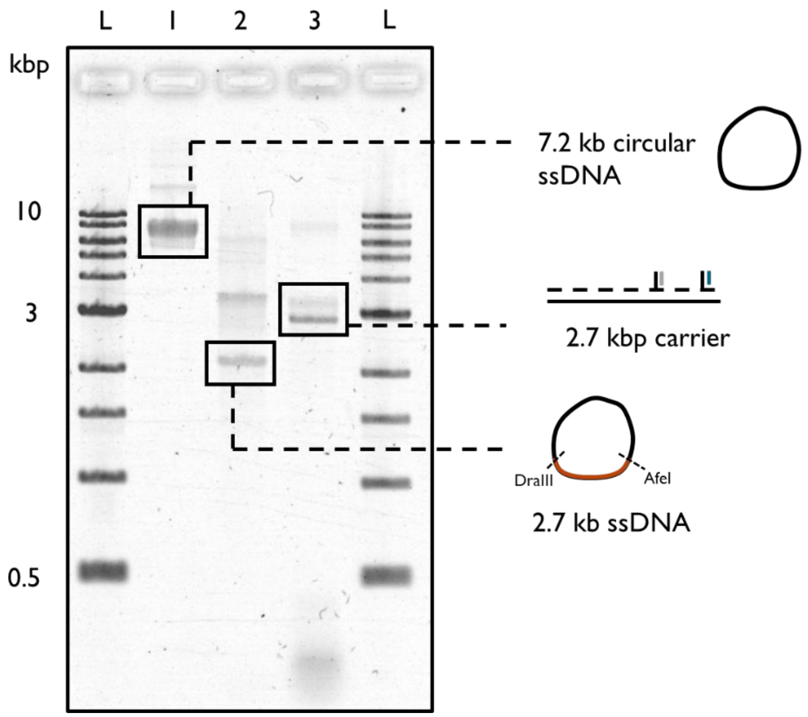

Figure S4. DNA carrier with short RNA assembly. **L** – 1 kbp DNA ladder (NEB). **1** – circular 7.2 kb ssDNA. **2** – Restriction cutting of 7.2 kb ssDNA to produce linear 2.7 kb ssDNA (DraIII-HF and AfeI). **3** – 2.7 kbp DNA carrier assembly containing the short RNA targets. Gel: 1% (w/v) agarose, 1 × TBE, 0.02% (w/v) sodium hypochlorite.

Figure S5.

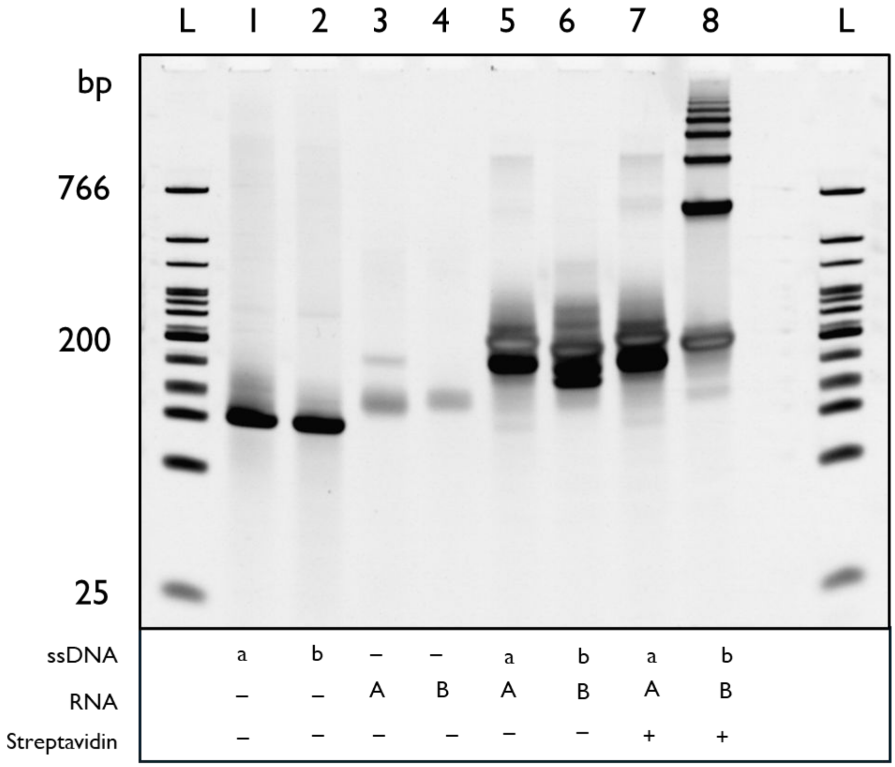

Figure S5. RNA:DNA duplex assembly and labeling. The characterization of the assay was done *via* gel electrophoresis in a 10% (v/v) native polyacrylamide gel in 1 × TBE**. L** – Low molecular weight ladder. **1** – ssDNA overhang *a* that binds to 2.7 kbp DNA carrier and the RNA *A* **2** – ssDNA overhang *b* that binds to 2.7 kbp DNA carrier and the RNA *B*. Sequence in Table S3. **3** – RNA *A*, does not contain biotinylated UTP. **4** – RNA *B* contains biotinylated UTP. **5** – RNA *A* + ssDNA overhang *a*. **6** – RNA *B* + ssDNA overhang *b* **7** – RNA *A* + ssDNA overhang *a* with monovalent streptavidin added. **8** – RNA *A* + ssDNA overhang *a* with monovalent streptavidin added. RNA:DNA duplexes are in excess.

Figure S6.

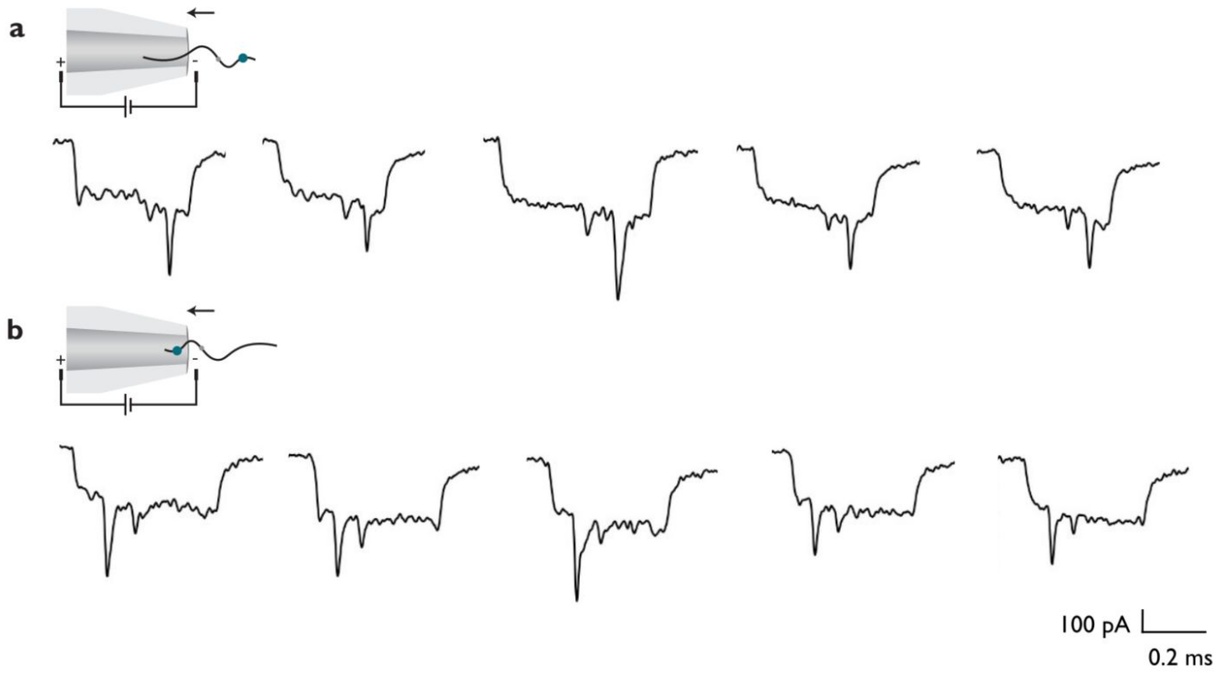

Figure S6. Raw current traces of DNA carrier with short RNA assembly translocating through the nanopore in the **(a)** 3′ to 5′ and the **(b)** 5′ to 3′ directions.

Figure S7.

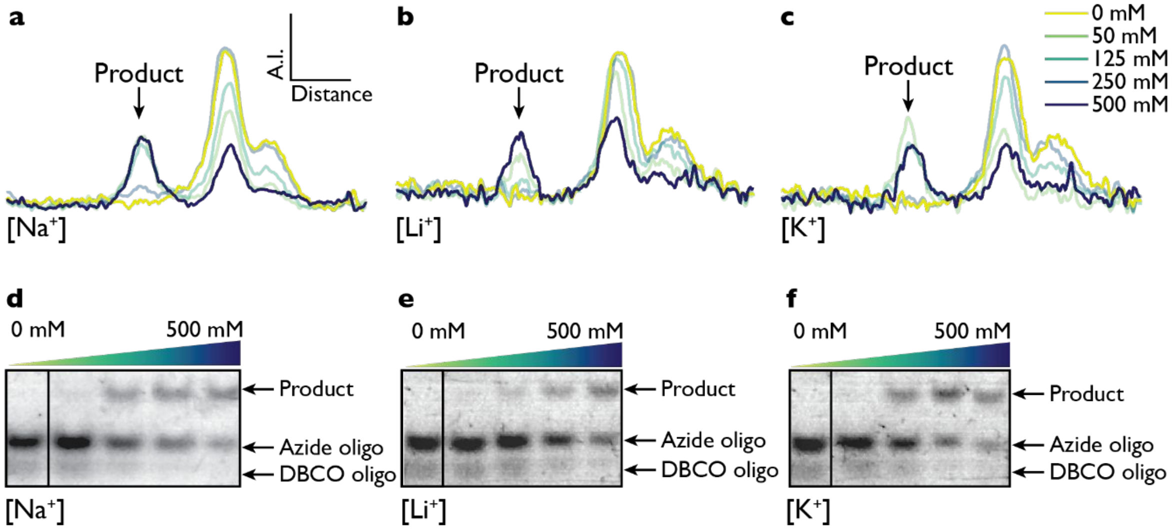

Figure S7. Effect of monovalent ion species on the template-free, catalyst-free click chemistry between azide containing oligo (26 nt) and DBCO-C6-oligo (15 nt). The reaction with variable concentrations of NaCl, LiCl, and KCl as shown in (**a, d**), (**b, e**), and (**c, f**), respectively. Arbitrary intensity (A.I.) of the lanes is plotted as a function of lane distance and the intensity plots of each lane are shown in (**a-c**) for the respective monovalent salt concentration at 0 mM, 50 mM, 125 mM, 250 mM, and 500 mM. The reaction proceeded with a 1:1 molar ratio of both oligonucleotides and was incubated at room temperature for 24 hr. Lane 1, depicting the no salt condition in (e) and (f) is a duplicate as the reaction was run on the same gel.

Figure S8.

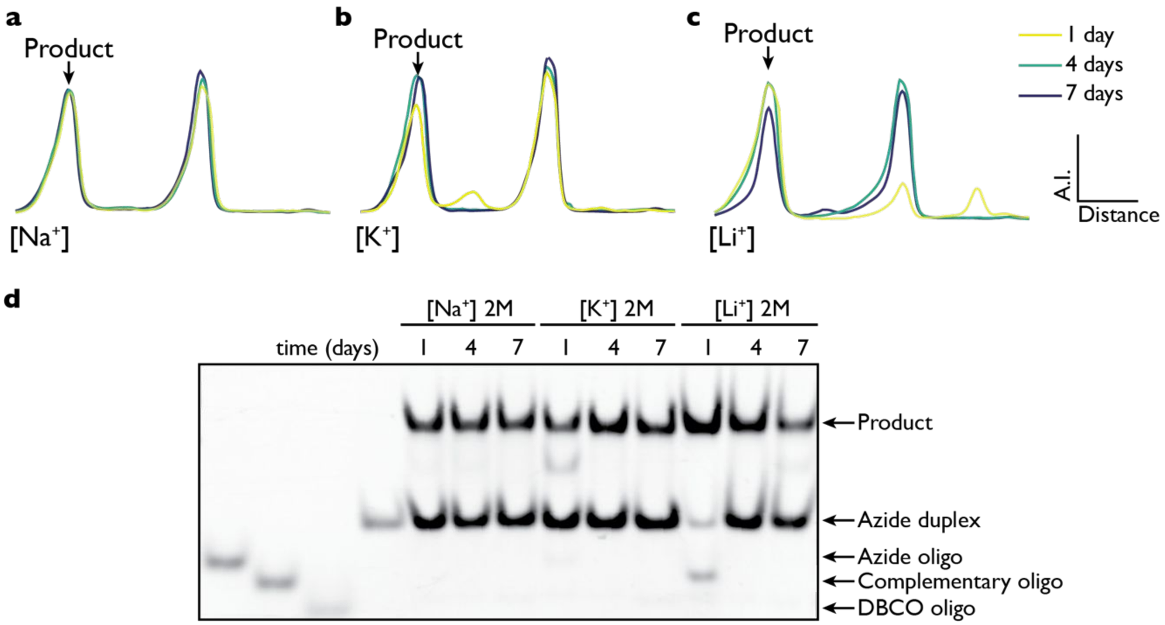

Figure S8. Effect of incubation in monovalent ion species on template-free, catalyst-free click chemistry between azide containing duplex (Azide duplex, 26 bp) and DBCO-PEG_4-oligo (15 nt). The reaction with variable incubation times at 37 °C in 2M NaCl, LiCl or KCl as shown in (**d**). The intensity plots of each lane are shown in (**a-c**) for the respective monovalent salt for 1, 4 and 7 day incubation time points.

Figure S9.

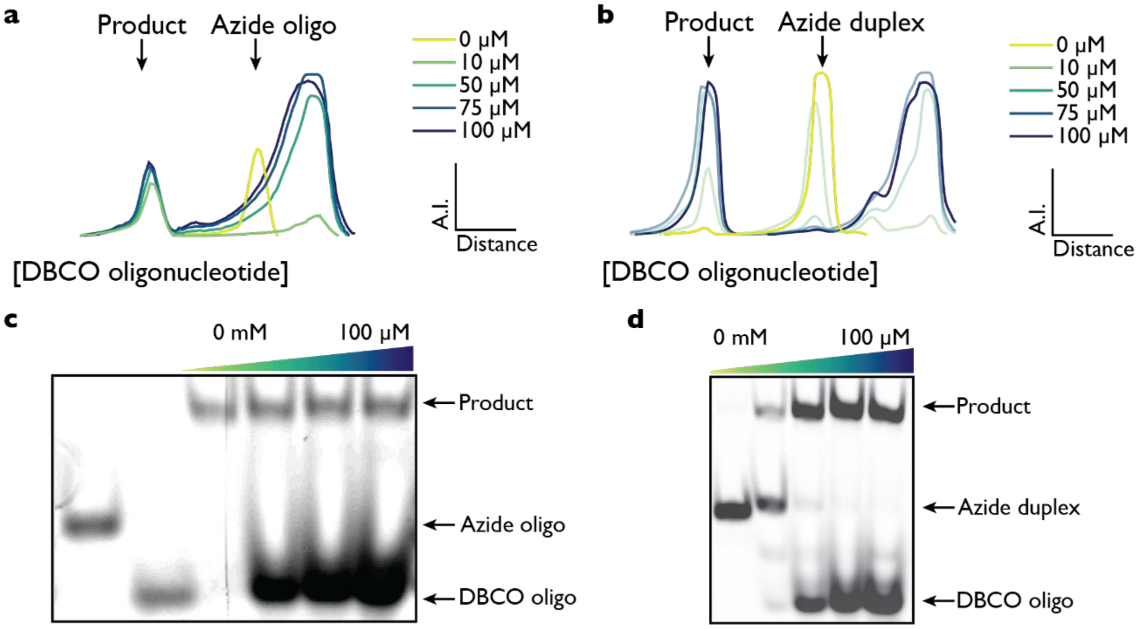

Figure S9. Template-free, catalyst-free click chemistry between DBCO-oligonucleotide and azide oligonucleotide or azide labelled duplex proceeds to completion within 1 hr under room temperature. Arbitrary intensity (A.I.) of the lanes is plotted as a function of lane distance. **(a)**, **(b)** depict the intensity plots for the reaction under varied concentrations DBCO-oligonucleotide. **(c)** and **(d)** show the corresponding (v/v) polyacrylamide gels in 1 × TBE. **(c)** Lanes 1-6 (left-right) show the azide oligonucleotide only (10 µg), DBCO oligo only (10 µg) or the reaction under varied concentrations of DBCO-oligo (10, 50, 75 and 100 µM). The reaction proceeded with the azide-oligonucleotide concentration of 10 µM and was incubated at RT for 1 hr. **(d)** Lanes 1-5 (left-right) show the reaction between the azide labeled duplex (10 µM) under varied concentrations of DBCO-oligo (0, 10, 50, 75 and 100 µM). The reaction proceeded with the azide labelled duplex concentration of 10 µM and was incubated at room temperature for 1 hr.

Figure S10.

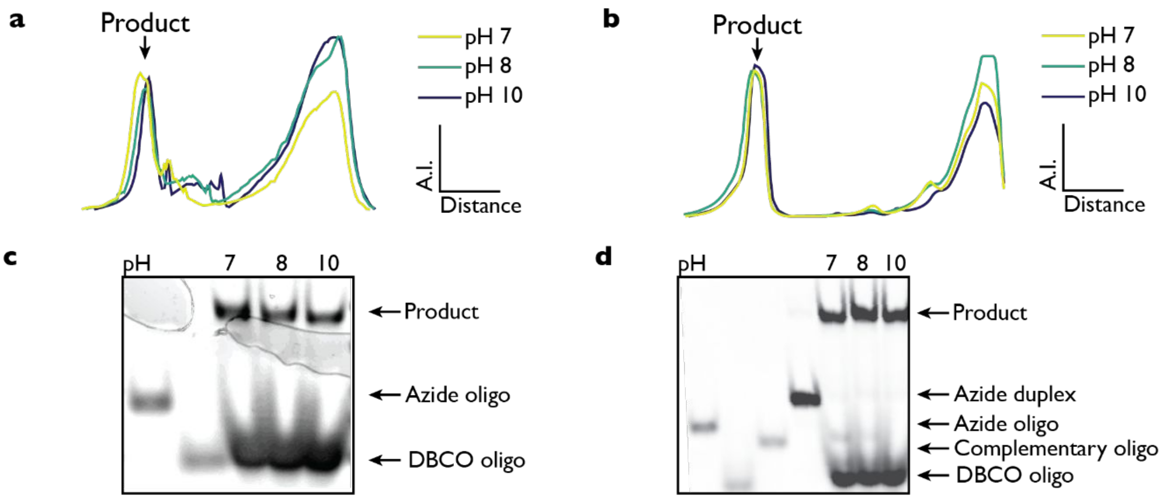

Figure S10. Template-free, catalyst-free click chemistry between DBCO-oligonucleotide and azide oligonucleotide or azide labelled duplex proceeds to completion within 1 hr under room temperature under different pH conditions. Arbitrary intensity (A.I.) of the lanes is plotted as a function of lane distance. **(a)**, **(b)** depict the intensity plots for the reaction under pH values of 7 (yellow), 8 (green) or 10 (purple). **(c)** and **(d)** show the corresponding (v/v) polyacrylamide gels in 1 × TBE. **(c)** Lanes 1-5 (left-right) show the azide labelled oligonucleotide only (10 µg), DBCO oligo only (10 µg) or the reaction under varied pH (7, 8, 10 respectively). The reaction proceeded with the azide-oligonucleotide or duplex concentration of 10 µM and was incubated at room temperature for 1 hr. **(d)** Lanes 1-5 (left-right) show the reaction between the azide labeled duplex (10 µM) under varied pH (7, 8, 10 respectively). The reactions shown proceeded with the azide-oligonucleotide or duplex concentration of 10 µM, DBCO- oligonucleotide concentration of 10 µM and were incubated at room temperature for 1 hr.

Figure S11.

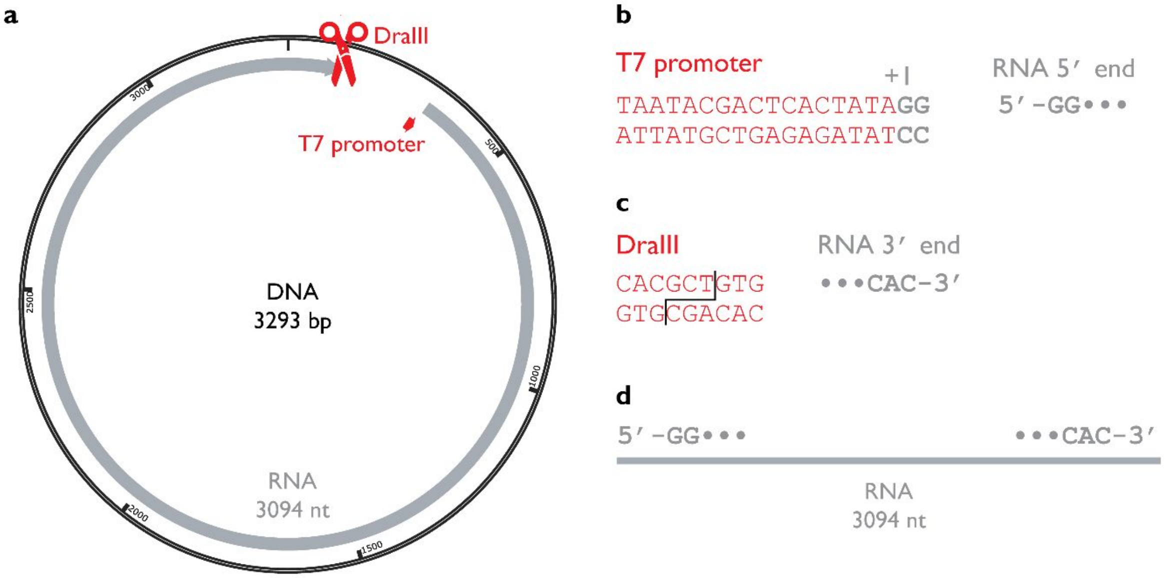

Figure S11. DNA plasmid is used for *in vitro* transcription of long modified RNA. (**a**) 3293 bp long dsDNA plasmid harboring T7 RNA polymerase promoter and DraIII restriction site used for *in vitro* transcription with azide-C3-UTP of RNA 3094 nt. (**b**) T7 RNA polymerase promoter sequence with two GG ensures that the first two nucleotides in RNA from 5`position are GG. (**c**) DraIII-HF was used for restriction digestion of plasmid to ensure that T7 RNA polymerase does not perform rolling circle transcription (*3*) and to define 3`end of RNA. (**d**) The start and the end of RNA sequence are shown here after *in vitro* transcription from the linearized DNA plasmid with DraIII.

Figure S12

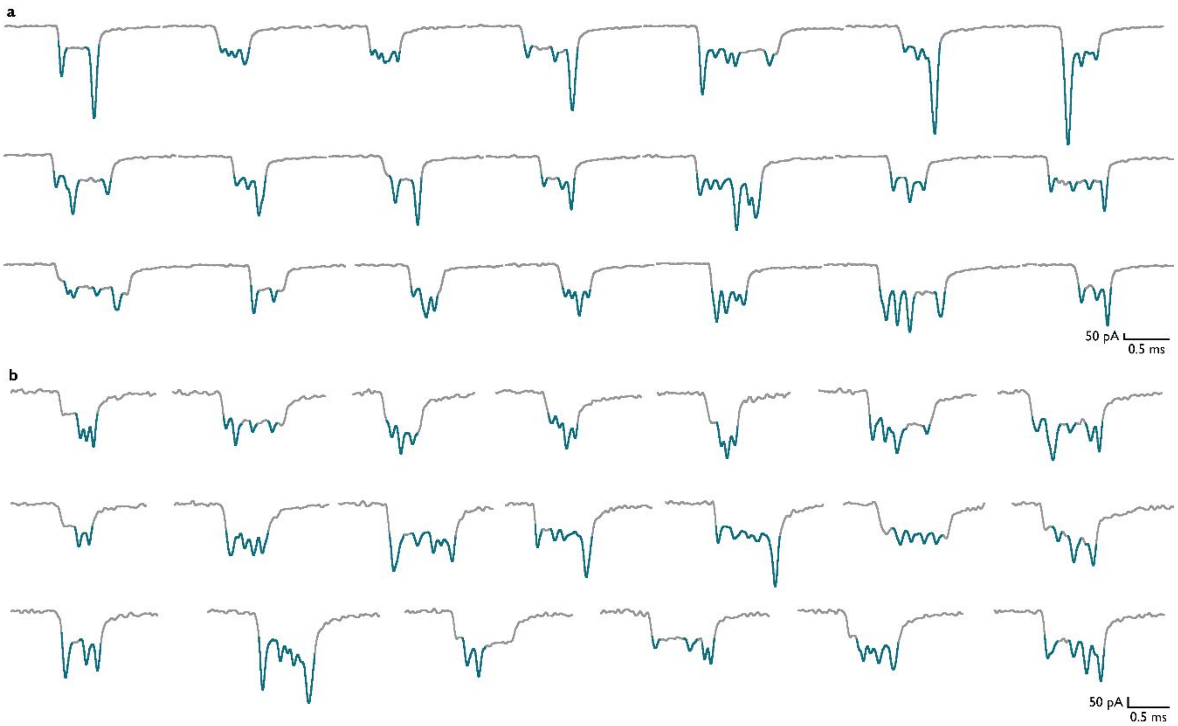

Figure S12. 3.1 kbp RNA ID additional nanopore events from two different nanopores in (**a**) and (**b**). Nanopores #2 and #3 were used for the measurements depicted in (**a**) and (**b**) respectively. The parameters for the nanopore data for these pores are shown in **Figure S15**.

Figure S13.

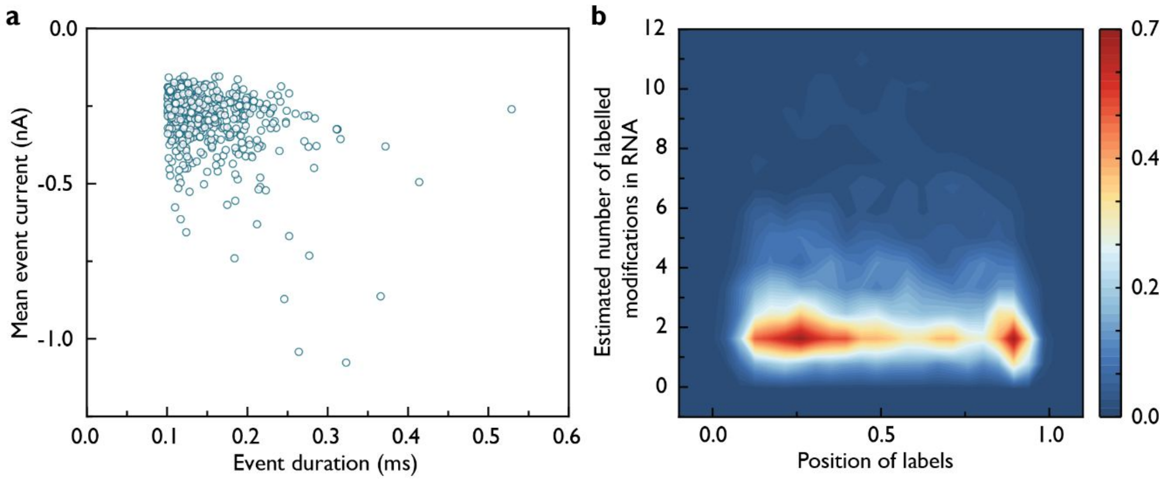

Figure S13. Scatter plot of mean event current versus event duration for the 3 kbp RNA nanostructure with the modifications (**a**). Density plot for the estimated number of labels versus their position along RNA molecule (**b**). The sample size was 1065 nanopore events.

Figure S14.

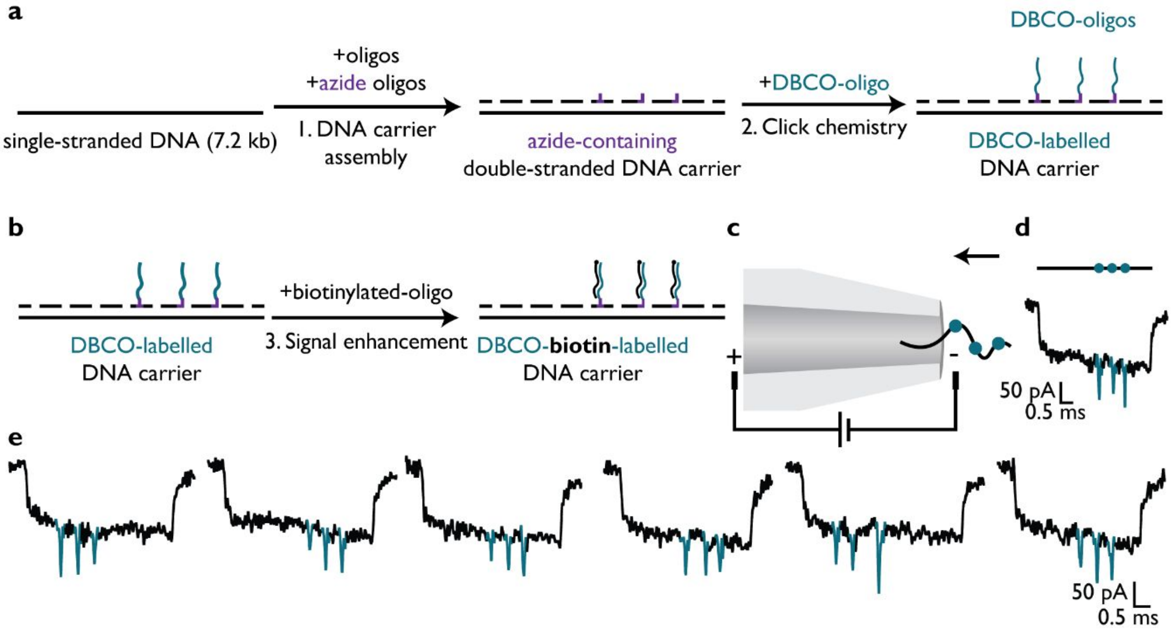

Figure S14. DNA carrier to assess azide oligonucleotide labeling with DBCO oligonucleotide. (**a**) Linearized single-stranded DNA from the M13 phage (7,228 nt) was employed as a scaffold for complementary oligonucleotides (**Table S8** and **S9**). Three specific azide modifications were positioned internally within the oligonucleotide, or at its 3′ end (indicated in purple). The 20 nt DBCO-oligonucleotide forms a triazole bond with azide groups through catalyst-free click chemistry (indicated in blue). (**b**) DBCO-labeled DNA carrier was hybridized with 3′-biotinylated oligonucleotides to produce a distinct structural signature, detectable by nanopore microscopy. (**c**) The glass nanopore microscope identified the DNA carrier, with an illustrative event shown in (**d**). This nanopore event features three downward spikes, each corresponding to a labeled site. Further nanopore events are depicted in (**e**).

Figure S15.

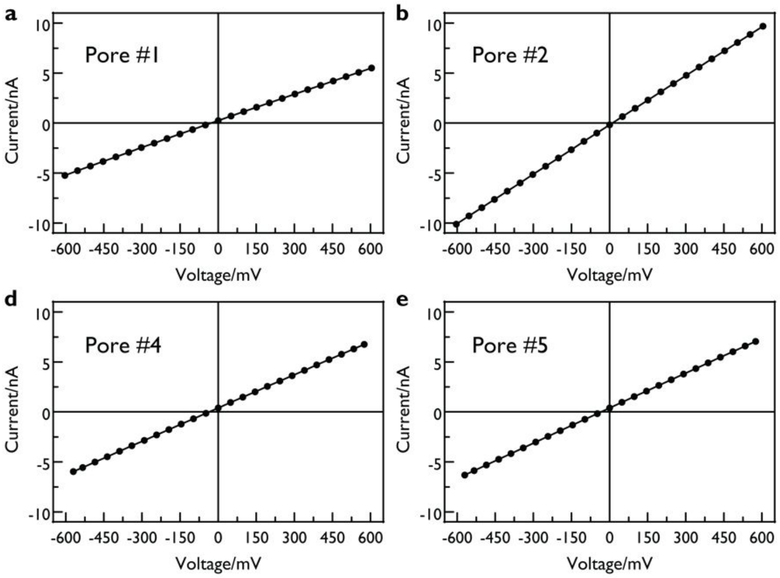

f

Pore #	Sample	Current (nA) at 600 mV	RMS noise (nA)
1	Fig.2	5.5	0.006
2	Fig.4	9.7	0.006
3	Fig.S12	7.3	0.006
4	Fig.S14	6.8	0.006
5	Fig.S14	7.1	0.006

Figure S15. Current/voltage (I/V) curves for all the nanopores used in this study. I/V curves are shown for nanopores used to measure events depicted in (**a**) Figure 2, (**b**) Figure 4, (**c**) Figure S12 and, (**d**) and (**e**) Figure S14. A summary table depicting the current and RMS noise for each nanopore is shown in (**f**).

Table S1.

Table S1. DNA oligonucleotide sequences used to produce DNA template *A* (*A2* is reverse complement of the *A1*) and DNA template *B* (*B2* is reverse complement of the *B1*) used for *in vitro* transcription and production of short RNAs. T7 RNA polymerase promoter in *cis* orientation is in bold.

DNA	Sequence (5′→ 3′)
A1	**TAATACGACTCACTATAGGG**ACTACAACACGACACGGACAACAACGACTACAAGAAGCACGACACGAGACCGACTACACC
A2	GGTGTAGTCGGTCTCGTGTCGTGCTTCTTGTAGTCGTTGTTGTCCGTGTCGTGTTGTAGTCCCTATAGTGAGTCGTATTA
B1	**TAATACGACTCACTATAGGG**ACAACAGACAAGACAACGAACAGCAGACTACACGAGCACGACAACGGAAAGGACAACAAG
B2	CTTGTTGTCCTTTCCGTTGTCGTGCTCGTGTAGTCTGCTGTTCGTTGTCTTGTCTGTTGTCCCTATAGTGAGTCGTATTA

Table S2.

Table S2. Sequence of ssM13 after restriction cutting with AfeI and DraIII-HF.

Sequence (5′→ 3′)
GCTCAATTACCCTCTGACTTTGTTCAGGGTGTTCAGTTAATTCTCCCGTCTAATGCGCT
TCCCTGTTTTTATGTTATTCTCTCTGTAAAGGCTGCTATTTTCATTTTTGACGTTAAAC
AAAAAATCGTTTCTTATTTGGATTGGGATAAATAATATGGCTGTTTATTTTGTAACTGG
CAAATTAGGCTCTGGAAAGACGCTCGTTAGCGTTGGTAAGATTCAGGATAAAATTGTAG
CTGGGTGCAAAATAGCAACTAATCTTGATTTAAGGCTTCAAAACCTCCCGCAAGTCGGG
AGGTTCGCTAAAACGCCTCGCGTTCTTAGAATACCGGATAAGCCTTCTATATCTGATTT
GCTTGCTATTGGGCGCGGTAATGATTCCTACGATGAAAATAAAAACGGCTTGCTTGTTC
TCGATGAGTGCGGTACTTGGTTTAATACCCGTTCTTGGAATGATAAGGAAAGACAGCCG
ATTATTGATTGGTTTCTACATGCTCGTAAATTAGGATGGGATATTATTTTTCTTGTTCA
GGACTTATCTATTGTTGATAAACAGGCGCGTTCTGCATTAGCTGAACATGTTGTTTATT
GTCGTCGTCTGGACAGAATTACTTTACCTTTTGTCGGTACTTTATATTCTCTTATTACT
GGCTCGAAAATGCCTCTGCCTAAATTACATGTTGGCGTTGTTAAATATGGCGATTCTCA
ATTAAGCCCTACTGTTGAGCGTTGGCTTTATACTGGTAAGAATTTGTATAACGCATATG
ATACTAAACAGGCTTTTTCTAGTAATTATGATTCCGGTGTTTATTCTTATTTAACGCCT
TATTTATCACACGGTCGGTATTTCAAACCATTAAATTTAGGTCAGAAGATGAAATTAAC
TAAAATATATTTGAAAAAGTTTTCTCGCGTTCTTTGTCTTGCGATTGGATTTGCATCAG
CATTTACATATAGTTATATAACCCAACCTAAGCCGGAGGTTAAAAAGGTAGTCTCTCAG
ACCTATGATTTTGATAAATTCACTATTGACTCTTCTCAGCGTCTTAATCTAAGCTATCG
CTATGTTTTCAAGGATTCTAAGGGAAAATTAATTAATAGCGACGATTTACAGAAGCAAG
GTTATTCACTCACATATATTGATTTATGTACTGTTTCCATTAAAAAAGGTAATTCAAAT
GAAATTGTTAAATGTAATTAATTTTGTTTTCTTGATGTTTGTTTCATCATCTTCTTTTG
CTCAGGTAATTGAAATGAATAATTCGCCTCTGCGCGATTTTGTAACTTGGTATTCAAAG
CAATCAGGCGAATCCGTTATTGTTTCTCCCGATGTAAAAGGTACTGTTACTGTATATTC
ATCTGACGTTAAACCTGAAAATCTACGCAATTTCTTTATTTCTGTTTTACGTGCAAATA
ATTTTGATATGGTAGGTTCTAACCCTTCCATTATTCAGAAGTATAATCCAAACAATCAG
GATTATATTGATGAATTGCCATCATCTGATAATCAGGAATATGATGATAATTCCGCTCC
TTCTGGTGGTTTCTTTGTTCCGCAAAATGATAATGTTACTCAAACTTTTAAAATTAATA
ACGTTCGGGCAAAGGATTTAATACGAGTTGTCGAATTGTTTGTAAAGTCTAATACTTCT
AAATCCTCAAATGTATTATCTATTGACGGCTCTAATCTATTAGTTGTTAGTGCTCCTAA
AGATATTTTAGATAACCTTCCTCAATTCCTTTCAACTGTTGATTTGCCAACTGACCAGA
TATTGATTGAGGGTTTGATATTTGAGGTTCAGCAAGGTGATGCTTTAGATTTTTCATTT
GCTGCTGGCTCTCAGCGTGGCACTGTTGCAGGCGGTGTTAATACTGACCGCCTCACCTC
TGTTTTATCTTCTGCTGGTGGTTCGTTCGGTATTTTTAATGGCGATGTTTTAGGGCTAT
CAGTTCGCGCATTAAAGACTAATAGCCATTCAAAAATATTGTCTGTGCCACGTATTCTT
ACGCTTTCAGGTCAGAAGGGTTCTATCTCTGTTGGCCAGAATGTCCCTTTTATTACTGG

```
TCGTGTGACTGGTGAATCTGCCAATGTAAATAATCCATTTCAGACGATTGAGCGTCAAA
ATGTAGGTATTTCCATGAGCGTTTTTCCTGTTGCAATGGCTGGCGGTAATATTGTTCTG
GATATTACCAGCAAGGCCGATAGTTTGAGTTCTTCTACTCAGGCAAGTGATGTTATTAC
TAATCAAAGAAGTATTGCTACAACGGTTAATTTGCGTGATGGACAGACTCTTTTACTCG
GTGGCCTCACTGATTATAAAAACACTTCTCAGGATTCTGGCGTACCGTTCCTGTCTAAA
ATCCCTTTAATCGGCCTCCTGTTTAGCTCCCGCTCTGATTCTAACGAGGAAAGCACGTT
ATACGTGCTCGTCAAAGCAACCATAGTACGCGCCCTGTAGCGGCGCATTAAGCGCGGCG
GGTGTGGTGGTTACGCGCAGCGTGACCGCTACACTTGCCAGCGCCCTAGCGCCCGCTCC
TTTCGCTTTCTTCCCTTCCTTTCTCGCCACGTTCGCCGGCTTTCCCCGTCAAGCTCTAA
ATCGGGGGCTCCCTTTAGGGTTCCGATTTAGTGCTTTACGGCACCTCGACCCCAAAAAA
CTTGATTTGGGTGATGGTTCACGTA
```

Table S3.

Table S3. DNA oligonucleotides used for the assembly of the 2.6 kbp DNA nanostructure and capture of short RNAs. The capture oligonucleotide sequences for short modified and unmodified RNA (A and B, respectively) are shown as *a* and *b*.

Oligo number	Sequence (5′→ 3′)
1	TACGTGAACCATCACCCAAATCAAGTTTTTTGGGGTCGAG
2	GTGCCGTAAAGCACTAAATCGGAACCCTAAAGGGAGCCCC
3	CGATTTAGAGCTTGACGGGGAAAGCCGGCGAACGTGGCGA
4	GAAAGGAAGGGAAGAAAGCGAAAGGAGCGGGCGCTAGGGC
5	GCTGGCAAGTGTAGCGGTCACGCTGCGCGTAACCACCACA
6	CCCGCCGCGCTTAATGCGCCGCTACAGGGCGCGTACTATG
7	GTTGCTTTGACGAGCACGTATAACGTGCTTTCCTCGTTAG
8	AATCAGAGCGGGAGCTAAACAGGAGGCCGATTAAAGGGAT
9	TTTAGACAGGAACGGTACGCCAGAATCCTGAGAAGTGTTT
10	TTATAATCAGTGAGGCCACCGAGTAAAAGAGTCTGTCCAT
11	CACGCAAATTAACCGTTGTAGCAATACTTCTTTGATTAGT
12	AATAACATCACTTGCCTGAGTAGAAGAACTCAAACTATCG
13	GCCTTGCTGGTAATATCCAGAACAATATTACCGCCAGCCA
14	TTGCAACAGGAAAAACGCTCATGGAAATACCTACATTTTG
15	ACGCTCAATCGTCTGAAATGGATTATTTACATTGGCAGAT
16	TCACCAGTCACACGACCAGTAATAAAAGGGACATTCTGGC
17	CAACAGAGATAGAACCCTTCTGACCTGAAAGCGTAAGAAT
18	ACGTGGCACAGACAATATTTTTGAATGGCTATTAGTCTTT
19	AATGCGCGAACTGATAGCCCTAAAACATCGCCATTAAAAA
20	TACCGAACGAACCACCAGCAGAAGATAAAACAGAGGTGAG
21	GCGGTCAGTATTAACACCGCCTGCAACAGTGCCACGCTGA
22	GAGCCAGCAGCAAATGAAAAATCTAAAGCATCACCTTGCT
23	GAACCTCAAATATCAAACCCTCAATCAATATCTGGTCAGT
24	TGGCAAATCAACAGTTGAAAGGAATTGAGGAAGGTTATCT
25	AAAATATCTTTAGGAGCACTAACAACTAATAGATTAGAGC
26	CGTCAATAGATAATACATTTGAGGATTTAGAAGTATTAGA
27	CTTTACAAACAATTCGACAACTCGTATTAAATCCTTTGCC
28	CGAACGTTATTAATTTTAAAAGTTTGAGTAACATTATCAT
29	TTTGCGGAACAAAGAAACCACCAGAAGGAGCGGAATTATC
30	ATCATATTCCTGATTATCAGATGATGGCAATTCATCAATA
31	TAATCCTGATTGTTTGGATTATACTTCTGAATAATGGAAG

**32**	GGTTAGAACCTACCATATCAAAATTATTTGCACGTAAAAC
**33**	AGAAATAAAGAAATTGCGTAGATTTTCAGGTTTAACGTCA
**34**	GATGAATATACAGTAACAGTACCTTTTACATCGGGAGAAA
**35**	CAATAACGGATTCGCCTGATTGCTTTGAATACCAAGTTAC
**36**	AAAATCGCGCAGAGGCGAATTATTCATTTCAATTACCTGA
**37**	GCAAAAGAAGATGATGAAACAAACATCAAGAAAACAAAAT
**38**	TAATTACATTTAACAATTTCATTTGAATTACCTTTTTTAA
**39**	TGGAAACAGTACATAAATCAAT
**40**	ATATGTGAGTGAATAACCTTGCT
**41**	TTTTCCCTTAGAATCCTTGAAAACATAGCGATAGCTTAGA
**42**	TTAAGACGCTGAGAAGAGTCAATAGTGAATTTATCAAAAT
**43**	CATAGGTCTGAGAGACTACCTTTTTAACCTCCGGCTTAGG
**44**	TTGGGTTATATAACTATATGTAAATGCTGATGCAAATCCA
**45**	ATCGCAAGACAAAGAACGCGAGAAAACTTTTTCAAATATA
**46**	TTTTAGTTAATTTCATCTTCTGACCTAAATTTAATGGTTT
**47**	GAAATACCGACCGTGTGATAAATAAGGCGTTAAATAAGAA
**48**	TAAACACCGGAATCATAATTACTAGAAAAAGCCTGTTTAG
**49**	TATCATATGCGTTATACAAATTCTTACCAGTATAAAGCCA
**50**	ACGCTCAACAGTAGGGCTTAATTGAGAATCGCCATATTTA
**51**	ACAACGCCAACATGTAATTTAGGCAGAGGCATTTTCGAGC
**52**	CAGTAATAAGAGAATATAAAGTACCGACAAAAGGT
**53**	AATAAACAACATGTTCAGCTAATGCAGAACGCGCCTGTTT
**54**	ATCAACAATAGATAAGTCCTGAACAAGAAAAATAATATCC
**55**	CATCCTAATTTACGAGCATGTAGAAACCAATCAATAATCG
**56**	GCTGTCTTTCCTTATCATTCCAAGAACGGGTATTAAACCA
**57**	AGTACCGCACTCATCGAGAACAAGCAAGCCGTTTTTATTT
**58**	TCATCGTAGGAATCATTACCGCGCCCAATAGCAAGCAAAT
**59**	CAGATATAGAAGGCTTATCCGGTATTCTAAGAACGCGAGG
**60**	CGTTTTAGCGAACCTCCCGACTTGCGGGAGGTTTTGAAGC
**61**	CTTAAATCAAGATTAGTTGCTATTTTGCACCCAGCTACAA
**62**	TTTTATCCTGAATCTTACCAACGCTAACGAGCGTCTTTCC
**63**	AGAGCCTAATTTGCCAGTTACAAAATAAACAGCCATATTA
**64**	TTTATCCCAATCCAAATAAGAAACGATTTTTTGTTTAACG
**65**	TCAAAAATGAAAATAGCAGCCTTTACAGAGAGAATAACAT
**66**	AAAAACAGGGAAGCGCATTAGACGGGAGAATTAACTGAAC
**67**	ACCCTGAACAAAGTCAGAGGGTAATTGAGC
**a**	TCTGTAAATCGTCGCTATTAATTAATTTGGTGTAGTCGGTCTCGTGTCG<br>TGCTTCTTGTAGTCGTTGTTGTCCGTGTCGTGTTGTAGTCCC

b AAAGTAATTCTGTCCAGACGACGACTTTCTTGTTGTCCTTTCCGTTGTCG
TGCTCGTGTAGTCTGCTGTTCGTTGTCTTGTCTGTTGTCCC

Table S4.

Table S4. Oligonucleotide sequences used for the click chemistry reactions in Figure 3, Figure 4, and Figure S14.

Oligonucleotide name	5′ → 3′ sequence	Length (nt)
Oligonucleotide with azide	GACCACTACAGT/iAzideN/GTAATCCTGACTT (iAzideN=T)	26
Complementary strand to oligonucleotide with azide	AAGTCAGGATTACAACTGTAGTGGTC	26
Oligonucleotide with DBCO-C6	ACCCATACTGAGTGA - C6 -DBCO	15
Oligonucleotide with DBCO-$(PEG)_4$	ACCCATACTGAGTGA - $(PEG)_4$ -DBCO	15
5′-biotin-TEG-oligonucleotide	/5bioTEG/TCACTCAGTATGGGT	15

Table S5.

Table S5. The 3293 bp plasmid sequence used for the *in vitro* transcription of long RNA with modifications (design is shown in **Figure S10**).

Sequence (5′→ 3′)
GCCCCTGCAGCCGAATTATATTATTTTTGCCAAATAATTTTTAACAAAAGCTCTGAAGT
CTTCTTCATTTAAATTCTTAGATGATACTTCATCTGGAAAATTGTCCCAATTAGTAGCA
TCACGCTGTGAGTAAGTTCTAAACCATTTTTTTATTGTTGTATTATCTCTAATCTTACT
ACTCGATGAGTTTTCGGTATTATCTCTATTTTTAACTTGGAGCAGGTTCCATTCATTGT
TTTTTTCATCATAGTGAATAAAATCAACTGCTTTAACACTTGTGCCTGAACACCATATC
CATCCGGCGTAATACGACTCACTATAGGGAGAGCGGCCGCCAGATCTTCCGGATGGCTC
GAGTTTTTCAGCAAGATCATGGTGCAGTGTAGCCGGGAATGCTGCTGCTGCTGCTG
CTGCTGCTGCTGCTGCTGCTGCTGCTGCTGCTGCTGCTGCTGCTGCTGCTGCTGCTGCT
GCTGCTGCTGCTGCTGCTGCTGCTGCTGCTGCTGCTGCTGCTGCTGCTGCTGCTGCTGC
TGCTGCTGCTGCTGCTGCTGCTGCTGCTGCTGCTGCTGCTGCTGCTGCTGCTGCTGCTG
CTGCTGCTGCTGCTGCTGCTGCTGCTGCTGCTGCTGCTGCTGCTGCTGCTGCTGCTGCT
GCTGCTGCTGCTGGGGGGATCACAGACCATTCTCGGCTTAAATCTTTCTAGAAGATCTC
CTACAATATTCTCAGCTGCCATGGAAAATCGATGTTCTTCTTTTATTCTCTCAAGATTT
TCAGGCTGTATATTAAAACTTATATTAAGAACTATGCTAACCACCTCATCAGGAACCGT
TGTAGGTGGCGTGGGTTTTCTTGGCAATCGACTCTCATGAAAACTACGAGCTAAATATT
CAATATGTTCCTCTTGACCAACTTTATTCTGCATTTTTTTTGAACGAGGTTTAGAGCAA
GCTTCAGGAAACTGAGACAGGAATTTTATTAAAAATTTAAATTTTGAAGAAAGTTCAGG
GTTAATAGCATCCATTTTTTTGCTTTGCAAGTTCCTCAGCATTCTTAACAAAGACGTCT
CTTTTGACATGTTTAAAGTTTAAACCTCCTGTGTGAAATTATTATCCGCTCATAATTCC
ACACATTATACGAGCCGGAAGCATAAAGTGTAAAGCCTGGGGTGCCTAATGAGTGAGCT
AACTCACATTAATTGCGTTGCGCTCACTGCCAATTGCTTTCCAGTCGGGAAACCTGTCG
TGCCAGCTGCATTAATGAATCGGCCAACGCGCGGGGAGAGGCGGTTTGCGTATTGGGCG
CTCTTCCGCTTCCTCGCTCACTGACTCGCTGCGCTCGGTCGTTCGGCTGCGGCGAGCGG
TATCAGCTCACTCAAAGGCGGTAATACGGTTATCCACAGAATCAGGGGATAACGCAGGA
AAGAACATGTGAGCAAAAGGCCAGCAAAAGGCCAGGAACCGTAAAAAGGCCGCGTTGCT
GGCGTTTTTCCATAGGCTCCGCCCCCCTGACGAGCATCACAAAAATCGACGCTCAAGTC
AGAGGTGGCGAAACCCGACAGGACTATAAAGATACCAGGCGTTTCCCCCTGGAAGCTCC
CTCGTGCGCTCTCCTGTTCCGACCCTGCCGCTTACCGGATACCTGTCCGCCTTTCTCCC
TTCGGGAAGCGTGGCGCTTTCTCATAGCTCACGCTGTAGGTATCTCAGTTCGGTGTAGG
TCGTTCGCTCCAAGCTGGGCTGTGTGCACGAACCCCCCGTTCAGCCCGACCGCTGCGCC
TTATCCGGTAACTATCGTCTTGAGTCCAACCCGGTAAGACACGACTTATCGCCACTGGC
AGCAGCCACTGGTAACAGGATTAGCAGAGCGAGGTATGTAGGCGGTGCTACAGAGTTCT
TGAAGTGGTGGCCTAACTACGGCTACACTAGAAGGACAGTATTTGGTATCTGCGCTCTG
CTGAAGCCAGTTACCTTCGGAAAAAGAGTTGGTAGCTCTTGATCCGGCAAACAAACCAC
CGCTGGTAGCGGTGGTTTTTTTGTTTGCAAGCAGCAGATTACGCGCAGAAAAAAAGGAT

CTCAAGAAGATCCTTTGATCTTTTCTACGGGGTCTGACGCTCAGTGGAACGAAAACTCA
CGTTAAGGGATTTTGGTCATGAGATTATCAAAAAGGATCTTCACCTAGATCCTTTTAAA
TTAAAAATGAAGTTTTAAATCAATCTAAAGTATATATGAGTAAACTTGGTCTGACAGTT
ACCAATGCTTAATCAGTGAGGCACCTATCTCAGCGATCTGTCTATTTCGTTCATCCATA
GTTGCCTGACTCCCCGTCGTGTAGATAACTACGATACGGGAGGGCTTACCATCTGGCCC
CAGTGCTGCAATGATACCGCGAGACCCACGCTCACCGGCTCCAGATTTATCAGCAATAA
ACCAGCCAGCCGGAAGGGCCGAGCGCAGAAGTGGTCCTGCAACTTTATCCGCCTCCATC
CAGTCTATTAATTGTTGCCGGGAAGCTAGAGTAAGTAGTTCGCCAGTTAATAGTTTGCG
CAACGTTGTTGCCATTGCTACAGGCATCGTGGTGTCACGCTCGTCGTTTGGTATGGCTT
CATTCAGCTCCGGTTCCCAACGATCAAGGCGAGTTACATGATCCCCCATGTTGTGCAAA
AAAGCGGTTAGCTCCTTCGGTCCTCCGATCGTTGTCAGAAGTAAGTTGGCCGCAGTGTT
ATCACTCATGGTTATGGCAGCACTGCATAATTCTCTTACTGTCATGCCATCCGTAAGAT
GCTTTTCTGTGACTGGTGAGTACTCAACCAAGTCATTCTGAGAATAGTGTATGCGGCGA
CCGAGTTGCTCTTGCCCGGCGTCAATACGGGATAATACCGCGCCACATAGCAGAACTTT
AAAAGTGCTCATCATTGGAAAACGTTCTTCGGGGCGAAAACTCTCAAGGATCTTACCGC
TGTTGAGATCCAGTTCGATGTAACCCACTCGTGCACCCAACTGATCTTCAGCATCTTTT
ACTTTCACCAGCGTTTCTGGGTGAGCAAAAACAGGAAGGCAAAATGCCGCAAAAAAGGG
AATAAGGGCGACACGGAAATGTTGAATACTCATACTCTTCCTTTTTCAATATTATTGAA
GCATTTATCAGGGTTATTGTCTCATGAGCGGATACATATTTGAATGTATTTAGAAAAAT
AAACAAATAGGGGTTCCGCGCACATTTCCCCGAAAAGTGCCACCTGACGTCTAAGAAAC
CATTATTATCATGACATTAACCTATAAAAATAGGCGTATCACGAGGCC

Table S6.

Table S6. The 3094 nt *in vitro* transcribed long RNA from the DraIII linearized plasmid as shown in Table S5 (design is in shown in Figure S11).

Sequence (5′→ 3′)
GGGAGAGCGGCCGCCAGAUCUUCCGGAUGGCUCGAGUUUUUCAGCAAGAUCAUGGUGCA
GUGUAGCCGGGAAUGCUGCUGCUGCUGCUGCUGCUGCUGCUGCUGCUGCUGCUGCUGCU
GCUGCUGCUGCUGCUGCUGCUGCUGCUGCUGCUGCUGCUGCUGCUGCUGCUGCUGCUGC
UGCUGCUGCUGCUGCUGCUGCUGCUGCUGCUGCUGCUGCUGCUGCUGCUGCUGCUGCUG
CUGCUGCUGCUGCUGCUGCUGCUGCUGCUGCUGCUGCUGCUGCUGCUGCUGCUGCUGCU
GCUGCUGCUGCUGCUGCUGCUGCUGCUGCUGCUGCUGCUGCUGCUGGGGGAUCACAGA
CCAUUCUCGGCUUAAAUCUUUCUAGAAGAUCUCCUACAAUAUUCUCAGCUGCCAUGGAA
AAUCGAUGUUCUUCUUUUAUUCUCUCAAGAUUUUCAGGCUGUAUAUUAAAACUUAUAUU
AAGAACUAUGCUAACCACCUCAUCAGGAACCGUUGUAGGUGGCGUGGGUUUUCUUGGCA
AUCGACUCUCAUGAAAACUACGAGCUAAAUAUUCAAUAUGUUCCUCUUGACCAACUUUA
UUCUGCAUUUUUUUUGAACGAGGUUUAGAGCAAGCUUCAGGAAACUGAGACAGGAAUUU
UAUUAAAAAUUUAAAUUUUGAAGAAAGUUCAGGGUUAAUAGCAUCCAUUUUUUGCUUUG
CAAGUUCCUCAGCAUUCUUAACAAAAGACGUCUCUUUUGACAUGUUUAAAGUUUAAACC
UCCUGUGUGAAAUUAUUAUCCGCUCAUAAUUCCACACAUUAUACGAGCCGGAAGCAUAA
AGUGUAAAGCCUGGGGUGCCUAAUGAGUGAGCUAACUCACAUUAAUUGCGUUGCGCUCA
CUGCCAAUUGCUUUCCAGUCGGGAAACCUGUCGUGCCAGCUGCAUUAAUGAAUCGGCCA
ACGCGCGGGGAGAGGCGGUUUGCGUAUUGGGCGCUCUUCCGCUUCCUCGCUCACUGACU
CGCUGCGCUCGGUCGUUCGGCUGCGGCGAGCGGUAUCAGCUCACUCAAAGGCGGUAAUA
CGGUUAUCCACAGAAUCAGGGGAUAACGCAGGAAAGAACAUGUGAGCAAAAGGCCAGCA
AAAGGCCAGGAACCGUAAAAAGGCCGCGUUGCUGGCGUUUUUCCAUAGGCUCCGCCCCC
CUGACGAGCAUCACAAAAAUCGACGCUCAAGUCAGAGGUGGCGAAACCCGACAGGACUA
UAAAGAUACCAGGCGUUUCCCCCUGGAAGCUCCCUCGUGCGCUCUCCUGUUCCGACCCU
GCCGCUUACCGGAUACCUGUCCGCCUUUCUCCCUUCGGGAAGCGUGGCGCUUUCUCAUA
GCUCACGCUGUAGGUAUCUCAGUUCGGUGUAGGUCGUUCGCUCCAAGCUGGGCUGUGUG
CACGAACCCCCCGUUCAGCCCGACCGCUGCGCCUUAUCCGGUAACUAUCGUCUUGAGUC
CAACCCGGUAAGACACGACUUAUCGCCACUGGCAGCAGCCACUGGUAACAGGAUUAGCA
GAGCGAGGUAUGUAGGCGGUGCUACAGAGUUCUUGAAGUGGUGGCCUAACUACGGCUAC
ACUAGAAGGACAGUAUUUGGUAUCUGCGCUCUGCUGAAGCCAGUUACCUUCGGAAAAAG
AGUUGGUAGCUCUUGAUCCGGCAAACAAACCACCGCUGGUAGCGGUGGUUUUUUUGUUU
GCAAGCAGCAGAUUACGCGCAGAAAAAAAGGAUCUCAAGAAGAUCCUUUGAUCUUUUCU
ACGGGGUCUGACGCUCAGUGGAACGAAAACUCACGUUAAGGGAUUUUGGUCAUGAGAUU
AUCAAAAAGGAUCUUCACCUAGAUCCUUUUAAAUUAAAAAUGAAGUUUUAAAUCAAUCU
AAAGUAUAUAUGAGUAAACUUGGUCUGACAGUUACCAAUGCUUAAUCAGUGAGGCACCU
AUCUCAGCGAUCUGUCUAUUUCGUUCAUCCAUAGUUGCCUGACUCCCCGUCGUGUAGAU
AACUACGAUACGGGAGGGCUUACCAUCUGGCCCCAGUGCUGCAAUGAUACCGCGAGACC

CACGCUCACCGGCUCCAGAUUUAUCAGCAAUAAACCAGCCAGCCGGAAGGGCCGAGCGC
AGAAGUGGUCCUGCAACUUUAUCCGCCUCCAUCCAGUCUAUUAAUUGUUGCCGGGAAGC
UAGAGUAAGUAGUUCGCCAGUUAAUAGUUUGCGCAACGUUGUUGCCAUUGCUACAGGCA
UCGUGGUGUCACGCUCGUCGUUUGGUAUGGCUUCAUUCAGCUCCGGUUCCCAACGAUCA
AGGCGAGUUACAUGAUCCCCCAUGUUGUGCAAAAAAGCGGUUAGCUCCUUCGGUCCUCC
GAUCGUUGUCAGAAGUAAGUUGGCCGCAGUGUUAUCACUCAUGGUUAUGGCAGCACUGC
AUAAUUCUCUUACUGUCAUGCCAUCCGUAAGAUGCUUUUCUGUGACUGGUGAGUACUCA
ACCAAGUCAUUCUGAGAAUAGUGUAUGCGGCGACCGAGUUGCUCUUGCCCGGCGUCAAU
ACGGGAUAAUACCGCGCCACAUAGCAGAACUUUAAAAGUGCUCAUCAUUGGAAAACGUU
CUUCGGGGCGAAAACUCUCAAGGAUCUUACCGCUGUUGAGAUCCAGUUCGAUGUAACCC
ACUCGUGCACCCAACUGAUCUUCAGCAUCUUUUACUUUCACCAGCGUUUCUGGGUGAGC
AAAAACAGGAAGGCAAAAUGCCGCAAAAAAGGGAAUAAGGGCGACACGGAAAUGUUGAA
UACUCAUACUCUUCCUUUUUCAAUAUUAUUGAAGCAUUUAUCAGGGUUAUUGUCUCAUG
AGCGGAUACAUAUUUGAAUGUAUUUAGAAAAAUAAACAAAUAGGGGUUCCGCGCACAUU
UCCCCGAAAAGUGCCACCUGACGUCUAAGAAACCAUUAUUAUCAUGACAUUAACCUAUA
AAAAUAGGCGUAUCACGAGGCCGCCCCUGCAGCCGAAUUAUAUUAUUUUUGCCAAAUAA
UUUUUAACAAAAGCUCUGAAGUCUUCUUCAUUUAAAUUCUUAGAUGAUACUUCAUCUGG
AAAAUUGUCCCAAUUAGUAGCAUCAC

Table S7.

Table S7. DNA oligonucleotides we used for the assembly of the 3 kbp RNA nanostructure i.e. RNA ID.

Oligo number	Sequence (5′→ 3′)
1	AGCGTGATGCTACTAATTGGGACAATTTTCCAGATGAA
2	GTATCATCTAAGAATTTAAATGAAGAAGACTTCAGAGC
3	TTTTGTTAAAAATTATTTGGCAAAAATAATATAATTCG
4	GCTGCAGGGGCGGCCTCGTGATACGCCTATTTTTATAG
5	GTTAATGTCATGATAATAATGGTTTCTTAGACGTCAGG
6	TGGCACTTTTCGGGGAAATGTGCGCGGAACCCCTATTT
7	GTTTATTTTTCTAAATACATTCAAATATGTATCCGCTC
8	ATGAGACAATAACCCTGATAAATGCTTCAATAATATTG
9	AAAAAGGAAGAGTATGAGTATTCAACATTTCCGTGTCG
10	CCCTTATTCCCTTTTTTGCGGCATTTTGCCTTCCTGTT
11	TTTGCTCACCCAGAAACGCTGGTGAAAGTAAAAGATGC
12	TGAAGATCAGTTGGGTGCACGAGTGGGTTACATCGAAC
13	TGGATCTCAACAGCGGTAAGATCCTTGAGAGTTTTCGC
14	CCCGAAGAACGTTTTCCAATGATGAGCACTTTTAAAGT
15	TCTGCTATGTGGCGCGGTATTATCCCGTATTGACGCCG
16	GGCAAGAGCAACTCGGTCGCCGCATACACTATTCTCAG
17	AATGACTTGGTTGAGTACTCACCAGTCACAGAAAAGCA
18	TCTTACGGATGGCATGACAGTAAGAGAATTATGCAGTG
19	CTGCCATAACCATGAGTGATAACACTGCGGCCAACTTA
20	CTTCTGACAACGATCGGAGGACCGAAGGAGCTAACCGC
21	TTTTTTGCACAACATGGGGGATCATGTAACTCGCCTTG
22	ATCGTTGGGAACCGGAGCTGAATGAAGCCATACCAAAC
23	GACGAGCGTGACACCACGATGCCTGTAGCAATGGCAAC
24	AACGTTGCGCAAACTATTAACTGGCGAACTACTTACTC
25	TAGCTTCCCGGCAACAATTAATAGACTGGATGGAGGCG
26	GATAAAGTTGCAGGACCACTTCTGCGCTCGGCCCTTCC
27	GGCTGGCTGGTTTATTGCTGATAAATCTGGAGCCGGTG
28	AGCGTGGGTCTCGCGGTATCATTGCAGCACTGGGGCCA
29	GATGGTAAGCCCTCCCGTATCGTAGTTATCTACACGAC
30	GGGGAGTCAGGCAACTATGGATGAACGAAATAGACAGA
31	TCGCTGAGATAGGTGCCTCACTGATTAAGCATTGGTAA
32	CTGTCAGACCAAGTTTACTCATATATACTTTAGATTGA

33	TTTAAAACTTCATTTTTAATTTAAAAGGATCTAGGTGA
34	AGATCCTTTTTGATAATCTCATGACCAAAATCCCTTAA
35	CGTGAGTTTTCGTTCCACTGAGCGTCAGACCCCGTAGA
36	AAAGATCAAAGGATCTTCTTGAGATCCTTTTTTTCTGC
37	GCGTAATCTGCTGCTTGCAAACAAAAAAACCACCGCTA
38	TCTTTTTCCGAAGGTAACTGGCTTCAGCAGAGCGCAGA
39	TACCAAATACTGTCCTTCTAGTGTAGCCGTAGTTAGGC
40	CACCACTTCAAGAACTCTGTAGCACCGCCTACATACCT
41	CGCTCTGCTAATCCTGTTACCAGTGGCTGCTGCCAGTG
42	GCGATAAGTCGTGTCTTACCGGGTTGGACTCAAGACGA
43	TAGTTACCGGATAAGGCGCAGCGGTCGGGCTGAACGGG
44	GGGTTCGTGCACACAGCCCAGCTTGGAGCGAACGACCT
45	ACACCGAACTGAGATACCTACAGCGTGAGCTATGAGAA
46	AGCGCCACGCTTCCCGAAGGGAGAAAGGCGGACAGGTA
47	TCCGGTAAGCGGCAGGGTCGGAACAGGAGAGCGCACGA
48	GGGAGCTTCCAGGGGGAAACGCCTGGTATCTTTATAGT
49	CCTGTCGGGTTTCGCCACCTCTGACTTGAGCGTCGATT
50	TTTGTGATGCTCGTCAGGGGGGCGGAGCCTATGGAAAA
51	ACGCCAGCAACGCGGCCTTTTTACGGTTCCTGGCCTTT
52	TGCTGGCCTTTTGCTCACATGTTCTTTCCTGCGTTATC
53	CCCTGATTCTGTGGATAACCGTATTACCGCCTTTGAGT
54	GAGCTGATACCGCTCGCCGCAGCCGAACGACCGAGCGC
55	AGCGAGTCAGTGAGCGAGGAAGCGGAAGAGCGCCCAAT
56	ACGCAAACCGCCTCTCCCCGCGCGTTGGCCGATTCATT
57	AATGCAGCTGGCACGACAGGTTTCCCGACTGGAAAGCA
58	ATTGGCAGTGAGCGCAACGCAATTAATGTGAGTTAGCT
59	CACTCATTAGGCACCCCAGGCTTTACACTTTATGCTTC
60	CGGCTCGTATAATGTGTGGAATTATGAGCGGATAATAA
61	TTTCACACAGGAGGTTTAAACTTTAAACATGTCAAAAG
62	AGACGTCTTTTGTTAAGAATGCTGAGGAACTTGCAAAG
63	CAAAAAATGGATGCTATTAACCCTGAACTTTCTTCAAA
64	ATTTAAATTTTTAATAAAATTCCTGTCTCAGTTTCCTG
65	AAGCTTGCTCTAAACCTCGTTCAAAAAAAATGCAGAAT
66	AAAGTTGGTCAAGAGGAACATATTGAATATTTAGCTCG
67	TAGTTTTCATGAGAGTCGATTGCCAAGAAAACCCACGC
68	CACCTACAACGGTTCCTGATGAGGTGGTTAGCATAGTT
69	CTTAATATAAGTTTTAATATACAGCCTGAAAATCTTGA
70	GAGAATAAAAGAAGAACATCGATTTTCCATGGCAGCTG

71	AGAATATTGTAGGAGATCTTCTAGAAAGATTTAAGCCG
72	AGAATGGTCTGTGATCCCCCCATTCCCGGCTACACTGC
73	ACCATGATCTTGCTGAAAAACTCGAGCC
74	ATCCGGAAGATCTGGCGGCCGCTCTCCC

Table S8.

Table S8. DNA oligonucleotide sequences we used for the assembly of the 7.2 kbp DNA nanostructure (Figure S14).

Oligo number	Sequence (5′→ 3′)
1	TTTTCGTAATCATGGTCATAGCTGTTTCCTGTGTGAAATTGTTATC
2	CGCTCACAATTCCACACAACATACGAGCCGGAAGCATA
3	AAGTGTAAAGCCTGGGGTGCCTAATGAGTGAGCTAACT
4	CACATTAATTGCGTTGCGCTCACTGCCCGCTTTCCAGT
5	CGGGAAACCTGTCGTGCCAGCTGCATTAATGAATCGGC
6	CAACGCGCGGGGAGAGGCGGTTTGCGTATTGGGCGCCA
7	GGGTGGTTTTTCTTTTCACCAGTGAGACGGGCAACAGC
8	TGATTGCCCTTCACCGCCTGGCCCTGAGAGAGTTGCAG
9	CAAGCGGTCCACGCTGGTTTGCCCCAGCAGGCGAAAAT
10	CCTGTTTGATGGTGGTTCCGAAATCGGCAAAATCCCTT
11	ATAAATCAAAAGAATAGCCCGAGATAGGGTTGAGTGTT
12	GTTCCAGTTTGGAACAAGAGTCCACTATTAAAGAACGT
13	GGACTCCAACGTCAAAGGGCGAAAAACCGTCTATCAGG
14	GCGATGGCCCACTACGTGAACCATCACCCAAATCAAGT
15	TTTTTGGGGTCGAGGTGCCGTAAAGCACTAAATCGGAA
16	CCCTAAAGGGAGCCCCCGATTTAGAGCTTGACGGGGAA
17	AGCCGGCGAACGTGGCGAGAAAGGAAGGGAAGAAAGCG
18	AAAGGAGCGGGCGCTAGGGCGCTGGCAAGTGTAGCGGT
19	CACGCTGCGCGTAACCACCACACCCGCCGCGCTTAATG
20	CGCCGCTACAGGGCGCGTACTATGGTTGCTTTGACGAG
21	CACGTATAACGTGCTTTCCTCGTTAGAATCAGAGCGGG
22	AGCTAAACAGGAGGCCGATTAAAGGGATTTTAGACAGG
23	AACGGTACGCCAGAATCCTGAGAAGTGTTTTTATAATC
24	AGTGAGGCCACCGAGTAAAAGAGTCTGTCCATCACGCA
25	AATTAACCGTTGTAGCAATACTTCTTTGATTAGTAATA
26	ACATCACTTGCCTGAGTAGAAGAACTCAAACTATCGGC
27	CTTGCTGGTAATATCCAGAACAATATTACCGCCAGCCA
28	TTGCAACAGGAAAAACGCTCATGGAAATACCTACATTT
29	TGACGCTCAATCGTCTGAAATGGATTATTTACATTGGC
30	AGATTCACCAGTCACACGACCAGTAATAAAAGGGACAT
31	TCTGGCCAACAGAGATAGAACCCTTCTGACCTGAAAGC
32	GTAAGAATACGTGGCACAGACAATATTTTTGAATGGCT
33	ATTAGTCTTTAATGCGCGAACTGATAGCCCTAAAACAT
34	CGCCATTAAAAATACCGAACGAACCACCAGCAGAAGAT
35	AAAACAGAGGTGAGGCGGTCAGTATTAACACCGCCTGC

36	AACAGTGCCACGCTGAGAGCCAGCAGCAAATGAAAAAT
37	CTAAAGCATCACCTTGCTGAACCTCAAATATCAAACCC
38	TCAATCAATATCTGGTCAGTTGGCAAATCAACAGTTGA
39	AAGGAATTGAGGAAGGTTATCTAAAATATCTTTAGGAG
40	CACTAACAACTAATAGATTAGAGCCGTCAATAGATAAT
41	ACATTTGAGGATTTAGAAGTATTAGACTTTACAAACAA
42	TTCGACAACTCGTATTAAATCCTTTGCCCGAACGTTAT
43	TAATTTTAAAAGTTTGAGTAACATTATCATTTTGCGGA
44	ACAAAGAAACCACCAGAAGGAGCGGAATTATCATCATA
45	TTCCTGATTATCAGATGATGGCAATTCATCAATATAAT
46	CCTGATTGTTTGGATTATACTTCTGAATAATGGAAGGG
47	TTAGAACCTACCATATCAAAATTATTTGCACGTAAAAC
48	AGAAATAAAGAAATTGCGTAGATTTTCAGGTTTAACGT
49	CAGATGAATATACAGTAACAGTACCTTTTACATCGGGA
50	GAAACAATAACGGATTCGCCTGATTGCTTTGAATACCA
51	AGTTACAAAATCGCGCAGAGGCGAATTATTCATTTCAA
52	TTACCTGAGCAAAAGAAGATGATGAAACAAACATCAAG
53	AAAACAAAATTAATTACATTTAACAATTTCATTTGAAT
54	TACCTTTTTTAATGGAAACAGTACATAAATCAATATAT
55	GTGAGTGAATAACCTTGCTTCTGTAAATCGTCGCTATT
56	AATTAATTTTCCCTTAGAATCCTTGAAAACATAGCGAT
57	AGCTTAGATTAAGACGCTGAGAAGAGTCAATAGTGAAT
58	TTATCAAAATCATAGGTCTGAGAGACTACCTTTTTAAC
59	CTCCGGCTTAGGTTGGGTTATATAACTATATGTAAATG
60	CTGATGCAAATCCAATCGCAAGACAAAGAACGCGAGAA
61	AACTTTTTCAAATATATTTTAGTTAATTTCATCTTCTG
62	ACCTAAATTTAATGGTTTGAAATACCGACCGTGTGATA
63	AATAAGGCGTTAAATAAGAATAAACACCGGAATCATAA
64	TTACTAGAAAAAGCCTGTTTAGTATCATATGCGTTATA
65	CAAATTCTTACCAGTATAAAGCCAACGCTCAACAGTAG
66	GGCTTAATTGAGAATCGCCATATTTAACAACGCCAACA
67	TGTAATTTAGGCAGAGGCATTTTCGAGCCAGTAATAAG
68	AGAATATAAAGTACCGACAAAAGGTAAAGTAATTCTGT
69	CCAGACGACGACAATAAACAACATGTTCAGCTAATGCA
70	GAACGCGCCTGTTTATCAACAATAGATAAGTCCTGAAC
71	AAGAAAAATAATATCCCATCCTAATTTACGAGCATGTA
72	GAAACCAATCAATAATCGGCTGTCTTTCCTTATCATTC
73	CAAGAACGGGTATTAAACCAAGTACCGCACTCATCGAG
74	AACAAGCAAGCCGTTTTTATTTTCATCGTAGGAATCAT
75	TACCGCGCCCAATAGCAAGCAAATCAGATATAGAAGGC
76	TTATCCGGTATTCTAAGAACGCGAGGCGTTTTAGCGAA

77	CCTCCCGACTTGCGGGAGGTTTTGAAGCCTTAAATCAA
78	GATTAGTTGCTATTTTGCACCCAGCTACAATTTTATCC
79	TGAATCTTACCAACGCTAACGAGCGTCTTTCCAGAGCC
80	TAATTTGCCAGTTACAAAATAAACAGCCATATTATTTA
81	TCCCAATCCAAATAAGAAACGATTTTTTGTTTAACGTC
82	AAAAATGAAAATAGCAGCCTTTACAGAGAGAATAACAT
83	AAAAACAGGGAAGCGCATTAGACGGGAGAATTAACTGA
84	ACACCCTGAACAAAGTCAGAGGGTAATTGAGCGCTAAT
85	ATCAGAGAGATAACCCACAAGAATTGAGTTAAGCCCAA
86	TAATAAGAGCAAGAAACAATGAAATAGCAATAGCTATC
87	TTACCGAAGCCCTTTTTAAGAAAAGTAAGCAGATAGCC
88	GAACAAAGTTACCAGAAGGAAACCGAGGAAACGCAATA
89	ATAACGGAATACCCAAAAGAACTGGCATGATTAAGACT
90	CCTTATTACGCAGTATGTTAGCAAACGTAGAAAATACA
91	TACATAAAGGTGGCAACATATAAAAGAAACGCAAAGAC
92	ACCACGGAATAAGTTTATTTTGTCACAATCAATAGAAA
93	ATTCATATGGTTTACCAGCGCCAAAGACAAAAGGGCGA
94	CATTCAACCGATTGAGGGAGGGAAGGTAAATATTGACG
95	GAAATTATTCATTAAAGGTGAATTATCACCGTCACCGA
96	CTTGAGCCATTTGGGAATTAGAGCCAGCAAAATCACCA
97	GTAGCACCATTACCATTAGCAAGGCCGGAAACGTCACC
98	AATGAAACCATCGATAGCAGCACCGTAATCAGTAGCGA
99	CAGAATCAAGTTTGCCTTTAGCGTCAGACTGTAGCGCG
100	TTTTCATCGGCATTTTCGGTCATAGCCCCCTTATTAGC
101	GTTTGCCATCTTTTCATAATCAAAATCACCGGAACCAG
102	AGCCACCACCGGAACCGCCTCCCTCAGAGCCGCCACCC
103	TCAGAACCGCCACCCTCAGAGCCACCACCCTCAGAGCC
104	GCCACCAGAACCACCACCAGAGCCGCCGCCAGCATTGA
105	CAGGAGGTTGAGGCAGGTCAGACGATTGGCCTTGATAT
106	TCACAAACAAATAAATCCTCATTAAAGCCAGAATGGAA
107	AGCGCAGTCTCTGAATTTACCGTTCCAGTAAGCGTCAT
108	ACATGGCTTTTGATGATACAGGAGTGTACTGGTAATAA
109	GTTTTAACGGGGTCAGTGCCTTGAGTAACAGTGCCCGT
110	ATAAACAGTTAATGCCCCCTGCCTATTTCGGAACCTAT
111	TATTCTGAAACATGAAAGTATTAAGAGGCTGAGACTCC
112	TCAAGAGAAGGATTAGGATTAGCGGGGTTTTGCTCAGT
113	ACCAGGCGGATAAGTGCCGTCGAGAGGGTTGATATAAG
114	TATAGCCCGGAATAGGTGTATCACCGTACTCAGGAGGT
115	TTAGTACCGCCACCCTCAGAACCGCCACCCTCAGAACC
116	GCCACCCTCAGAGCCACCACCCTCATTTTCAGGGATAG
117	CAAGCCCAATAGGAACCCATGTACCGTAACACTGAGTT

118	TCGTCACCAGTACAAACTACAACGCCTGTAGCATTCCA
119	CAGACAGCCCTCATAGTTAGCGTAACGATCTAAAGTTT
120	TGTCGTCTTTCCAGACGTTAGTAAATGAATTTTCTGTA
121	TGGGATTTTGCTAAACAACTTTCAACAGTTTCAGCGGA
122	GTGAGAATAGAAAGGAACAACTAAAGGAATTGCGAATA
123	ATAATTTTTTCACGTTGAAAATCTCCAAAAAAAAGGCT
124	CCAAAAGGAGCCTTTAATTGTATCGGTTTATCAGCTTG
125	CTTTCGAGGTGAATTTCTTAAACAGCTTGATACCGATA
126	GTTGCGCCGACAATGACAACAACCATCGCCCACGCATA
127	ACCGATATATTCGGTCGCTGAGGCTTGCAGGGAGTTAA
128	AGGCCGCTTTTGCGGGATCGTCACCCTCAGCAGCGAAA
129	GACAGCATCGGAACGAGGGTAGCAACGGCTACAGAGGC
130	TTTGAGGACTAAAGACTTTTTCATGAGGAAGTTTCCAT
131	TAAACGGGTAAAATACGTAATGCCACTACGAAGGCACC
132	AACCTAAAACGAAAGAGGCAAAAGAATACACTAAAACA
133	CTCATCTTTGACCCCCAGCGATTATACCAAGCGCGAAA
134	CAAAGTACAACGGAGATTTGTATCATCGCCTGATAAAT
135	TGTGTCGAAATCCGCGACCTGCTCCATGTTACTTAGCC
136	GGAACGAGGCGCAGACGGTCAATCATAAGGGAACCGAA
137	CTGACCAACTTTGAAAGAGGACAGATGAACGGTGTACA
138	GACCAGGCGCATAGGCTGGCTGACCTTCATCAAGAGTA
139	ATCTTGACAAGAACCGGATATTCATTACCCAAATCAAC
140	GTAACAAAGCTGCTCATTCAGTGAATAAGGCTTGCCCT
141	GACGAGAAACACCAGAACGAGTAGTAAATTGGGCTTGA
142	GATGGTTTAATTTCAACTTTAATCATTGTGAATTACCT
143	TATGCGATTTTAAGAACTGGCTCATTATACCAGTCAGG
144	ACGTTGGGAAGAAAAATCTACGTTAATAAAACGAACTA
145	ACGGAACAACATTATTACAGGTAGAAAGATTCATCAGT
146	TGAGATTTAGGAATACCACATTCAACTAATGCAGATAC
147	ATAACGCCAAAAGGAATTACGAGGCATAGTAAGAGCAA
148	CACTATCATAACCCTCGTTTACCAGACGACGATAAAAA
149	CCAAAATAGCGAGAGGCTTTTGCAAAAGAAGTTTTGCC
150	AGAGGGGGTAATAGTAAAATGTTTAGACTGGATAGCGT
151	CCAATACTGCGGAATCGTCATAAATATTCATTGAATCC
152	CCCTCAAATGCTTTAAACAGTTCAGAAAACGAGAATGA
153	CCATAAATCAAAAATCAGGTCTTTACCCTGACTATTAT
154	AGTCAGAAGCAAAGCGGATTGCATCAAAAAGATTAAGA
155	GGAAGCCCGAAAGACTTCAAATATCGCGTTTTAATTCG
156	AGCTTCAAAGCGAACCAGACCGGAAGCAAACTCCAACA
157	GGTCAGGATTAGAGAGTACCTTTAATTGCTCCTTTTGA
158	TAAGAGGTCATTTTTGCGGATGGCTTAGAGCTTAATTG

159	CTGAATATAATGCTGTAGCTCAACATGTTTTAAATATG
160	CAACTAAAGTACGGTGTCTGGAAGTTTCATTCCATATA
161	ACAGTTGATTCCCAATTCTGCGAACGAGTAGATTTAGT
162	TTGACCATTAGATACATTTCGCAAATGGTCAATAACCT
163	GTTTAGCTATATTTTCATTTGGGGCGCGAGCTGAAAAG
164	GTGGCATCAATTCTACTAATAGTAGTAGCATTAACATC
165	CAATAAATCATACAGGCAAGGCAAAGAATTAGCAAAAT
166	TAAGCAATAAAGCCTCAGAGCATAAAGCTAAATCGGTT
167	GTACCAAAAACATTATGACCCTGTAATACTTTTGCGGG
168	AGAAGCCTTTATTTCAACGCAAGGATAAAAATTTTTAG
169	AACCCTCATATATTTTAAATGCAATGCCTGAGTAATGT
170	GTAGGTAAAGATTCAAAAGGGTGAGAAAGGCCGGAGAC
171	AGTCAAATCACCATCAATATGATATTCAACCGTTCTAG
172	CTGATAAATTAATGCCGGAGAGGGTAGCTATTTTTGAG
173	AGATCTACAAAGGCTATCAGGTCATTGCCTGAGAGTCT
174	GGAGCAAACAAGAGAATCGATGAACGGTAATCGTAAAA
175	CTAGCATGTCAATCATATGTACCCCGGTTGATAATCAG
176	AAAAGCCCCAAAAACAGGAAGATTGTATAAGCAAATAT
177	TTAAATTGTAAACGTTAATATTTTGTTAAAATTCGCAT
178	TAAATTTTTGTTAAATCAGCTCATTTTTTAACCAATAG
179	GAACGCCATCAAAAATAATTCGCGTCTGGCCTTCCTGT
180	AGCCAGCTTTCATCAACATTAAATGTGAGCGAGTAACA
181	ACCCGTCGGATTCTCCGTGGGAACAAACGGCGGATTGA
182	CCGTAATGGGATAGGTCACGTTGGTGTAGATGGGCGCA
183	TCGTAACCGTGCATCTGCCAGTTTGAGGGGACGACGAC
184	AGTATCGGCCTCAGGAAGATCGCACTCCAGCCAGCTTT
185	CCGGCACCGCTTCTGGTGCCGGAAACCAGGCAAAGCGC
186	CATTCGCCATTCAGGCTGCGCAACTGTTGGGAAGGGCG
187	ATCGGTGCGGGCCTCTTCGCTATTACGCCAGCTGGCGA
188	AAGGGGGATGTGCTGCAAGGCGATTAAGTTGGGTAACG
189	CCAGGGTTTTCCCAGTCACGACGTTGTAAAACGACGGC
190	CAGTGCCAAGCTTGCATGCCTGCAGGTCGACTCTAGAGGATCTTTT

Table S9.

Table S9. Oligonucleotides which were substituted in place of standard oligos from **Table S8** when forming double-stranded DNA nanostructure 7.2 kbp for the click chemistry reaction between azide-containing DNA nanostructure and DBCO-C6-oligonucleotide. Oligo 97 with azide was used in place of oligonucleotide number 97, oligo 121 with azide was used in place of oligonucleotide number 121, oligo 142 with azide was used in place of oligonucleotide number 142. The azide-containing oligos were ordered from IDT and the respective 3′ or internal azide modifications are highlighted.

Oligo number	Sequence (5′→ 3′)	Length (nt)
Oligonucleotide 97 with azide	GTAGCACCATTACCAT/iAzideN/AGCAAGGCCGGAAA CGTCACC (iAzideN=T)	38
Oligonucleotide 121 with azide	TGGGATTTTGCTAAACAACTTTCAACAGTTT CAGCGGA/3AzideN/	38
Oligonucleotide 142 with azide	GATGGTTTAATTTCAACTTTAATCATTGTGA ATTACCT/3AzideN/	38
